# *ERCC5* , *HES6* and *RORA* are potential diagnostic markers of coronary artery disease

**DOI:** 10.1002/2211-5463.13469

**Published:** 2022-08-07

**Authors:** Zhifeng Bai, Yuanyuan Luo, Linyun Tian

**Affiliations:** ^1^ Second Department of Cardiovascular Medicine The First People's Hospital of Shangqiu Shangqiu City China; ^2^ Department of Endocrinology and Metabolism, The First Affiliated Hospital of Zhengzhou University Zhengzhou University Zhengzhou China; ^3^ Department of Cardiology, The First People's Hospital of Yunnan Province The Affiliated Hospital of Kunming University of Science and Technology Kunming China

**Keywords:** bioinformatics, coronary heart disease, diagnostic marker, *ERCC5*, *HES6*, *RORA*

## Abstract

The mortality rate of patients with coronary artery disease (CAD) increases year by year, and the age of onset is decreasing, primarily because of the lack of an efficient and convenient diagnostic method for CAD. In the present study, we aimed to detect CAD‐correlated biomarkers and the regulatory pathways involved through weighted co‐expression network analysis. The microarray data originated from 93 CAD patients and 48 controls within the Gene Expression Omnibus (GEO) database. The gene network was implemented by weighted gene co‐expression network analysis, and the genes were observed to fall into a range of modules. We took the intersection of genes in the modules most correlated with CAD with the differentially expressed genes of CAD, which were identified by applying the limma package. Lasso regression and support vector machine recursive feature elimination algorithms were used to determine CAD candidate signature genes. The biomarkers for diagnosing CAD were detected by validating candidate signature gene diagnostic capabilities (receiver operating characteristic curves) based on data sets from GEO. Three modules were selected, and 26 vital genes were identified. Eight of these genes were reported as the optimal candidate features in terms of CAD diagnosis. Through receiver operating characteristic curve analysis, we identified three genes (*ERCC5*, *HES6* and *RORA*; area under the curve > 0.8) capable of distinguishing CAD from the control, and observed that these genes are correlated with the immune response. In summary, *ERCC5*, *HES6* and *RORA* may have potential for diagnosis of CAD.

## Graphical abstract

The present study aimed to detect coronary artery disease (CAD)‐correlated biomarkers and the regulatory pathways through weighted co‐expression network analysis; we identified three genes (i.e. *ERCC5*, *HES6* and *RORA*) capable of distinguishing CAD from the control and report that immune‐correlated gene sets were correlated with each other, thereby presenting novel possibilities for early CAD diagnosis and prevention.

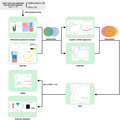

**Abbreviations**
AUC: area under the curve
CAD: coronary artery disease
DEG: differentially expressed gene
GEO: Gene Expression Omnibus
GS: gene significance
GSEA: gene set enrichment analysis
LASSO: lasso regression
MM: module membership
qRT‐PCR: quantitative real‐time PCR
ROC: receiver operating characteristic
SVM‐RFE: support vector machine recursive feature elimination
WGCNA: weighted gene co‐expression network analysis

Coronary atherosclerotic heart disease (CAD) has been recognized as a primary cause of morbidity and mortality in octogenarians, imposing a huge economic burden on society [1]. As coronary interventional techniques have been applied clinically, and statins and antiplatelet therapy have been developing, the clinical prognosis of CAD patients [2] is significantly facilitated. Nevertheless, the mortality rate of CAD patients continues to increase year by year, and the age of onset is becoming increasingly younger [3], primarily because of the lack of an efficient and convenient diagnostic method for CAD. Thus, specific early diagnostic biomarkers in CAD patients should be explored.

The pathogenesis of CAD has been reported to be significantly correlated with some biological processes (e.g. lipid disorder, vascular inflammation, oxidative stress and vascular endothelial dysfunction) [4]. In addition, the immune system is primarily involved in the pathophysiology of cardiovascular disease [5]. Monocytes and macrophages can fall to a proinflammatory phenotype and a healing phenotype in extreme cases. The interaction and balance between the mentioned two phenotypes are critical to atherosclerosis [6, 7]. According to a survey of subjects with premature CAD, older CAD patients were found with higher levels of T cell, macrophage and T regulatory cell infiltration into coronary plaques [8].

Currently, bioinformatics plays a decisive role in gene function research, protein structure prediction, precision medicine, drug design and forensic identification. In the present study, *ERCC5*, *HES6* and *RORA* are identified as the diagnostic markers of CAD. Specifically, the *ERCC5* is an important endonuclease in the NER pathway. The mutation of *ERCC5* can induce abnormal cell proliferation and differentiation, which would promote tumorigenesis. However, the role of *ERCC5* in CAD remains unclear [9]. *HES6* plays an indirect regulatory role in the Notch signaling pathway. However, the function and mechanism of *HES6* in the occurrence and progression of CAD have not been clarified. The expression of *RORA* can be widely observed in the heart, lung, liver and other tissues [10]. *RORA* has the main function of encoding NR1 (thyroid hormone) and regulating circadian rhythms. It shall be noted that circadian rhythms are essential for the heart to maintain a healthy state. Moreover, it has been revealed that the high expression of *RORA* correlates with acute myocardial infarction, which may be an independent risk factor for acute myocardial infarction [11]. Furthermore, in the present study, these genes are involved in the development of CAD through some immune‐related pathways, including humoral immune response, regulation of immune response, positive regulation of immune response, immune response‐regulating signaling pathway, immune effector process, regulation immune system process, immune system process and regulation of immune system process. Therefore, the diagnostic biomarkers related to CAD can be identified in the present study through the exploration, screening, as well as analysis of genes related to CAD. Besides, the potential immune pathways were investigated in an attempt to provide insights and data support for early diagnosis and prevention of CAD.

## Materials and methods

### Source of datasets

Two CAD‐correlated gene expression profiles (i.e. GSE113079 and GSE23561) were identified in the Gene Expression Omnibus (GEO) database (https://www.ncbi.nlm.nih.gov/geo) and determined after searching, both comprising microarray data from CAD and normal samples. In GSE113079, 93 CAD patients and 48 normal subjects were reported. Moreover, in GSE23561, six CAD patients and nine normal subjects were included to validate diagnostic genes. The chips from the data sets were peripheral blood samples.

### Screening for differentially expressed genes (DEGs)

Given the comparison of expression values between CAD samples and normal samples in the GSE113079 data set, the R package *limma* [12] was adopted to screen for DEGs. The screening conditions for DEGs were *P* < 0.05 and ¦log_2_FC¦ ≥ 1.0. The results are illustrated separately by the heat map and volcano map drawn with the r software package (R Foundation for Statistical Computing, Vienna, Austria).

### Weighted gene co‐expression network analysis (WGCNA)

The R package *WGCNA* was employed to build a co‐expression network for all genes with mean expression values over 1 in the CAD samples and normal samples in the GSE113079 data set. Here, CAD and control served as the clinical traits for WGCNA. A cluster analysis on all samples in the GSE113079 data set was first conducted to determine the presence of outlying samples. Next, the clinical data of CAD and normal samples were introduced to the cluster diagram to build a sample cluster‐clinical trait heatmap. Power parameters ranging from 1 to 20 were filtered out with the pickSoftThreshold function. An appropriate soft threshold power of 6 was determined because it satisfied scale‐free *R* ^2^ = 0.85 at the minimum power value. Subsequently, when MEDissThres was set to 0.5, a hybrid dynamic clipping tree algorithm was exploited to segment and merge similar modules.

Furthermore, the correlation between the merged modules and clinical features of CAD was calculated to identify vital modules significantly correlated with clinical features of CAD. The correlation coefficient and the *P* value are presented in the heatmap. Gene significance (GS) was defined as the correlation between gene expression and the respective trait. Module membership (MM) was defined as the correlation between gene expression and the characteristic genes of the respective module. The genes were selected from the vital modules satisfying ¦MM¦ > 0.8 and ¦G¦ > 0.2 as hub genes for subsequent studies. Through the online website jvenn (http://jvenn.toulouse.inra.fr/app/example.html), the analysis exploited the intersection of the differential genes and Hub genes acquired from the screening above to identify the genes most correlated with CAD progression, which were termed vital genes.

### Least absolute shrinkage and selection operator (LASSO) and support vector machine model‐recursive feature elimination (SVM‐RFE) algorithms

The LASSO algorithm and SVM‐RFE algorithm were comprehensively applied for screening the biomarkers of CAD. First, 141 samples randomly fell to the training set (*n* = 99) and the validation set (*n* = 42) at 7 : 3. The glmnet package in r was employed for the LASSO regression algorithm with penalty parameter regulation through 10‐fold cross‐validation. When lambda.min was 0.004955, it would be the optimal diagnostic model for CAD. The SVM‐RFE algorithm was implemented with the e1071 software package of r. As the number of genes began to vary, the error rate of the prediction of the optimal points of CAD samples and normal samples reached 0.035, exhibiting an accuracy of 0.965, thereby complying with the most appropriate characteristics. The diagnostic markers of CAD were finally determined by overlapping the signature genes of the two algorithms.

### Receiver operating characteristic (ROC) curve analysis

To assess the effect exerted by candidate genes in the diagnosis of CAD, ROC curve analysis was conducted in rstudio (RStudio, Boston, MA, USA) for genes selected by the multivariate model in combination [13] with the proc package. Genes with an area under the curve (AUC) > 0.8 acted as the diagnostic genes for CAD. Moreover, the diagnostic significance of the mentioned diagnostic genes was validated in the GSE23561 data set.

### Enrichment analysis by the Gene Set Enrichment Analysis (GSEA)

To further explore the pathways involved with diagnostic genes in CAD, CAD patients were separated in the GSE113079 data set into high and low expression groups by complying with the expression of diagnostic genes. The GSEA was conducted to verify whether a set of priori defined genes achieved significant differential expression high expression group and the low expression group in the enrichment collected by MSigDB [14]. In this study, the ordered list of all genes was first generated by drawing upon the correlation with the expression of diagnostic genes. Next, the GSEA was adopted to assess significant differences between the results of the high expression group and the low expression group. *P* < 0.05 was considered statistically significant with respect to enrichment.

### Extraction of total RNA from blood and quantitative real‐time PCR (qRT‐PCR)

To more specifically validate the expression of diagnostic genes in CAD, we first observed the expression of diagnostic genes in CAD and normal samples using the GEO data set, and then collected blood samples from CAD patients (*n* = 10) and normal subjects (*n* = 10) from the First People's Hospital of Yunnan Province for validation. The studies involving human participants were reviewed and approved by the Ethics Committee of the First People's Hospital of Yunnan Province. The patients provided written informed consent to participate in this study. The collection and processing of all samples were performed by strictly complying with the regulations of the Ethics Committee of the First People's Hospital of Yunnan Province. Specific information on the patients is provided in Table S1. The study methodologies conformed to the standards set by the Declaration of Helsinki.

The overall RNA from blood was extracted with the Trizol reagent method, and the quality and purity of the extracted RNA were tested based on a nucleic acid protein instrument. The extracted RNA was reverse transcribed into cDNA to detect the relative expression of the target gene mRMA in accordance with the SureScript‐First‐strand‐cDNA‐synthesis‐kit instructions (Genecopoeia, Rockville, MD, USA). Primers were synthesized following the sequences (Table 1) transferred to Optimus Biological Company (Beijing, China).

**Table 1:** Primer sequences.

Genes	Forward	Reverse
*ERCC5*	GCCTGGAACCTCTCCTAAAA	CGAAATACCGCTGACAAAAT
*RORA*	CACCTACTCCTGTCCTCGTC	AGTCTGCCTTACTCCCCTCA
*HES6*	TGCTGGAGCTGACGGTGCGG	GGGGGGCTGGGTATTGGGGA
*GAPDH*	CCTTCCGTGTTCCTACCCC	GCCCAAGATGCCCTTCAGT

qRT‐PCR reactions were performed by employing the BlazeTaq™ SYBR® Green qPCR Mix2.0 kit (Genecopoeia) with the reaction systems oulined below: cDNA, 4 μL; 5 × BlazeTaq qPCR Mix, 2 μL; PCR forward primer (2 μm), 0.5 μL; PCR reverse primer (2 μm), 0.5 μL; ddH_2_O (RNase/DNase free) 1 μL for 10 μL in total. The reaction program consisted of denaturation at 95 °C for 30 s; denaturation at 95 °C for 10 s, annealing at 60 °C for 20 s, for 40 cycles, as well as extension 72 °C fr 30 s. The *C*_T_ value of the respective gene was counted, and the relative expression of the gene was calculated based on the $2^{-\Delta \Delta C_{t}}$ method by applying GAPDH as the internal reference gene.

### Statistical analysis

All the bioinformatics analyses in the present study were conducted with the r software package. The expression heatmap of vital genes in CAD patients and the healthy controls was constructed using the pheatmap package. To compare the expression differences of vital genes between CAD patients and the healthy controls, the rank‐sum test was performed with the R language ggpubr package. *P* < 0.05 was considered statistically significant.

## Results

### Screening for DEGs of CAD

**Fig. 1:**
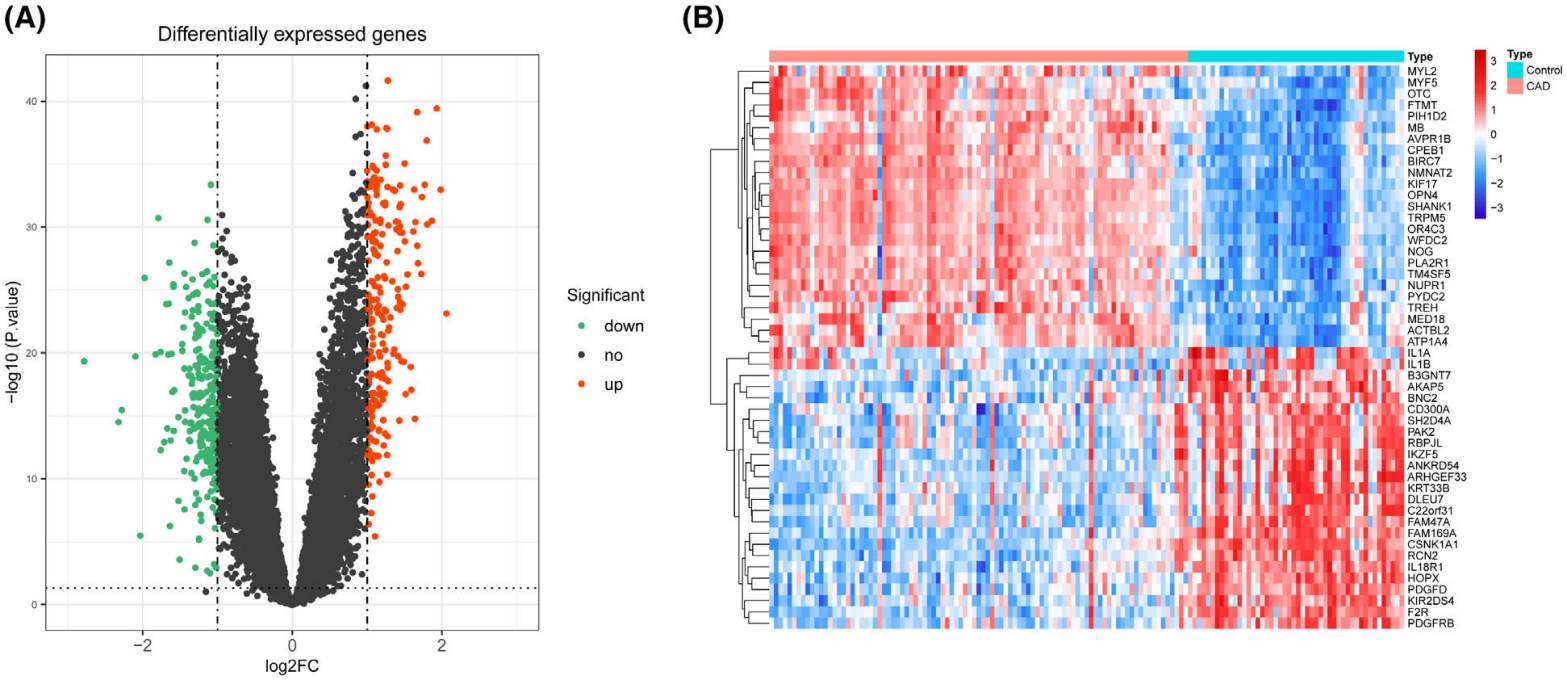
(A) Volcano plot of differentially expressed genes (dots in green and red represent genes with significant differential expression; dots in red indicate that their gene expression was up‐regulated in the CAD samples and dots in green indicate that the gene was down‐regulated in the CAD samples). (B) Differentially expressed gene heatmap (the top 25 up‐ and the top 25 down‐regulated genes) (each small square indicates a gene, with red indicating up‐regulated and blue indicating down‐regulated).

By employing the limma package to analyze the differential expression of the GSE113079 data set, 480 DEGs were overall obtained (i.e. 220 up‐regulated genes and 260 down‐regulated genes) (Table S2). The volcano plot presents the DEGs screened by threshold (*P* < 0.05 and ¦log_2_FC¦ ≥ 1.0) (Fig. 1A). Figure 1B illustrates the expression of the top 25 up‐ and the top 25 down‐regulated genes ranked by *P* value and log_2_FC value in the respective sample.

### Screening of highly relevant critical modules in CAD

**Fig. 2:**
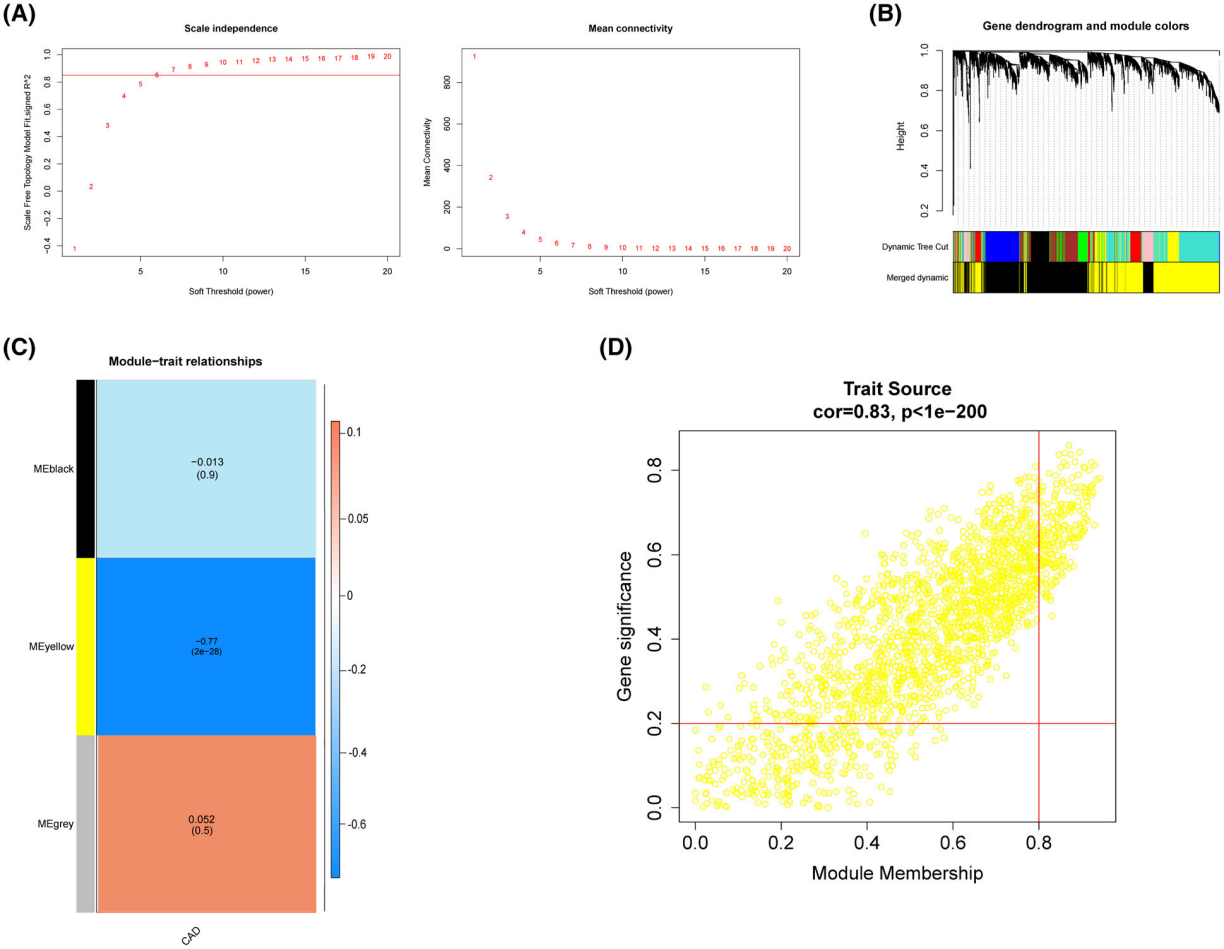
CAD highly relevant critical modules. (A) Soft threshold screening [the longitudinal axis of the left figure is scale‐freefitindex (i.e. signedR2) and the square of the correlation coefficient, indicating how close the network is to the scale‐free distribution; the longitudinal axis of the right figure represents all gene adjacency functions in the corresponding gene module]. (B) Merge plots of modules. (C) Heat map of modules versus traits. (D) Correlation analysis between module genes and traits.

To explore the vital modules most relevant to the onset of CAD, WGCNA was performed on samples in the GSE113079 data set. No outliers were detected in the clusters, and so a hierarchical cluster tree was constructed with 93 CAD samples and 48 normal samples (Fig. S1A,B). Next, a power of β = 6 was selected as the soft threshold to ensure that intergenic interactions maximally conformed to the scale‐free network (Fig. 2A). As a result, the dynamic shearing tree algorithm was adopted to segment into nine modules, and three modules were finally obtained after merging similar modules (Fig. 2B). Subsequently, WGCNA was exploited to correlate the respective module with all available clinical information in the GSE113079 data set by calculating CAD feature correlations for each module. After all modules were screened for significant correlations with CAD progression, the MEyellow module was reported with the maximal correlation coefficient among all selected modules (correlation = −0.77, *P* < 0.01) (Fig. 2C). As revealed from the correlation analysis between module genes and CAD traits, the correlation coefficient between the MEyellow genes and module traits reached 0.83 (*P* < 0.05). In total, 1917 genes in the MEyellow module, of which 207 genes satisfied GS > 0.2 and MM > 0.8, served as the vital module genes for subsequent analysis (Fig. 2D).

### Screening of vital genes for CAD

**Fig. 3:**
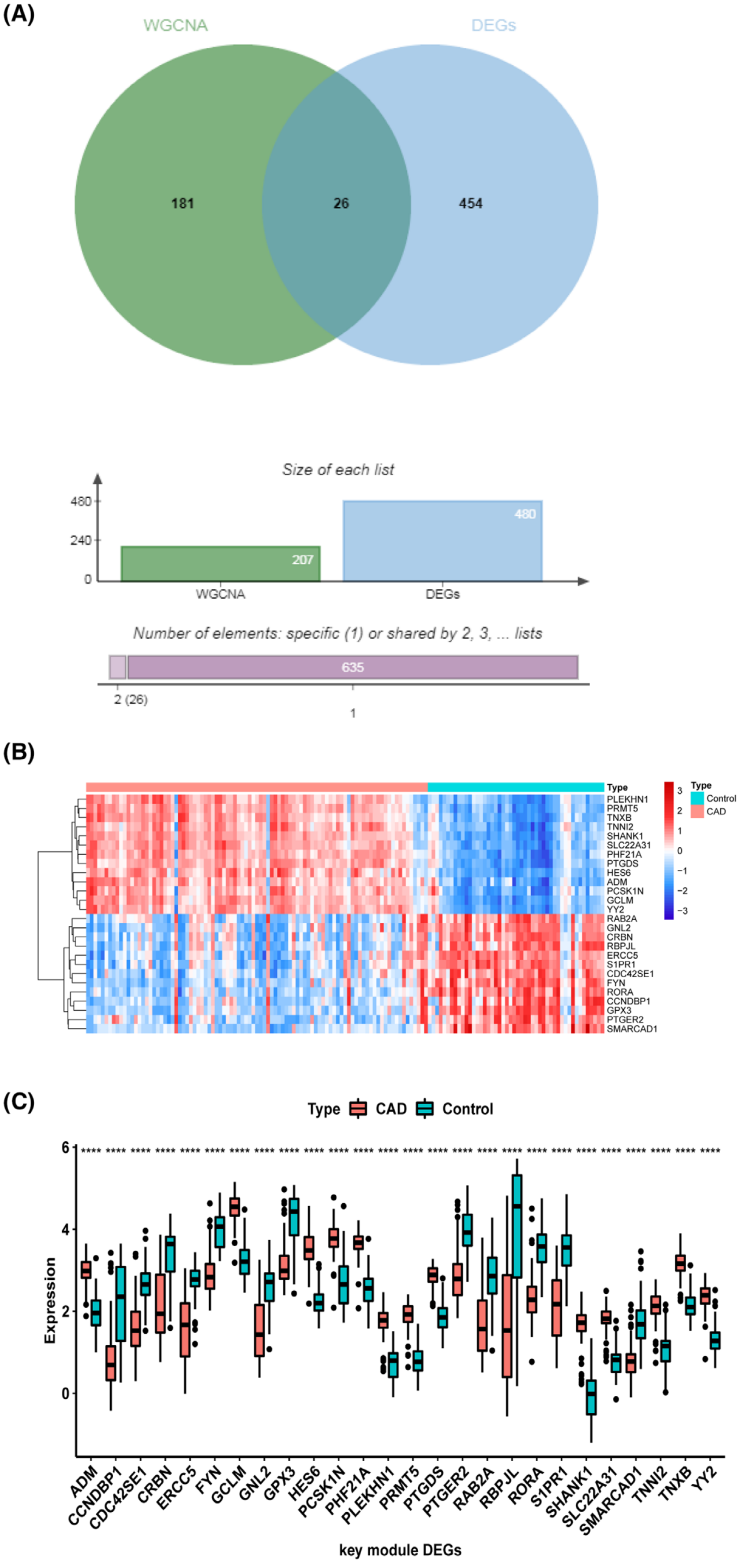
Vital genes for CAD. (A) Module key gene screen (blue represents genes differentially expressed between CAD/control and green represents WGCNA acquisition of CAD vital module genes). (B) Heat map of expression patterns of module vital genes between CAD patients and controls. (C) Box plots of key gene expression of modules (blue represents control samples and red represents CAD samples). *P < 0.0001.

We intersected the differentially expressed genes between CAD and control with the CAD‐correlated vital module genes obtained by WGCNA. Then, 26 genes in total were termed vital genes for CAD (Fig. 3A).

Figure 3B demonstrates the expression of vital genes in CAD patients and healthy controls. By using the rank sum test, the significant differences of vital genes between CAD patients and healthy controls were compared and presented using box plots (*P* < 0.0001) (Fig. 3C).

### Screening for CAD diagnostic markers

**Fig. 4:**
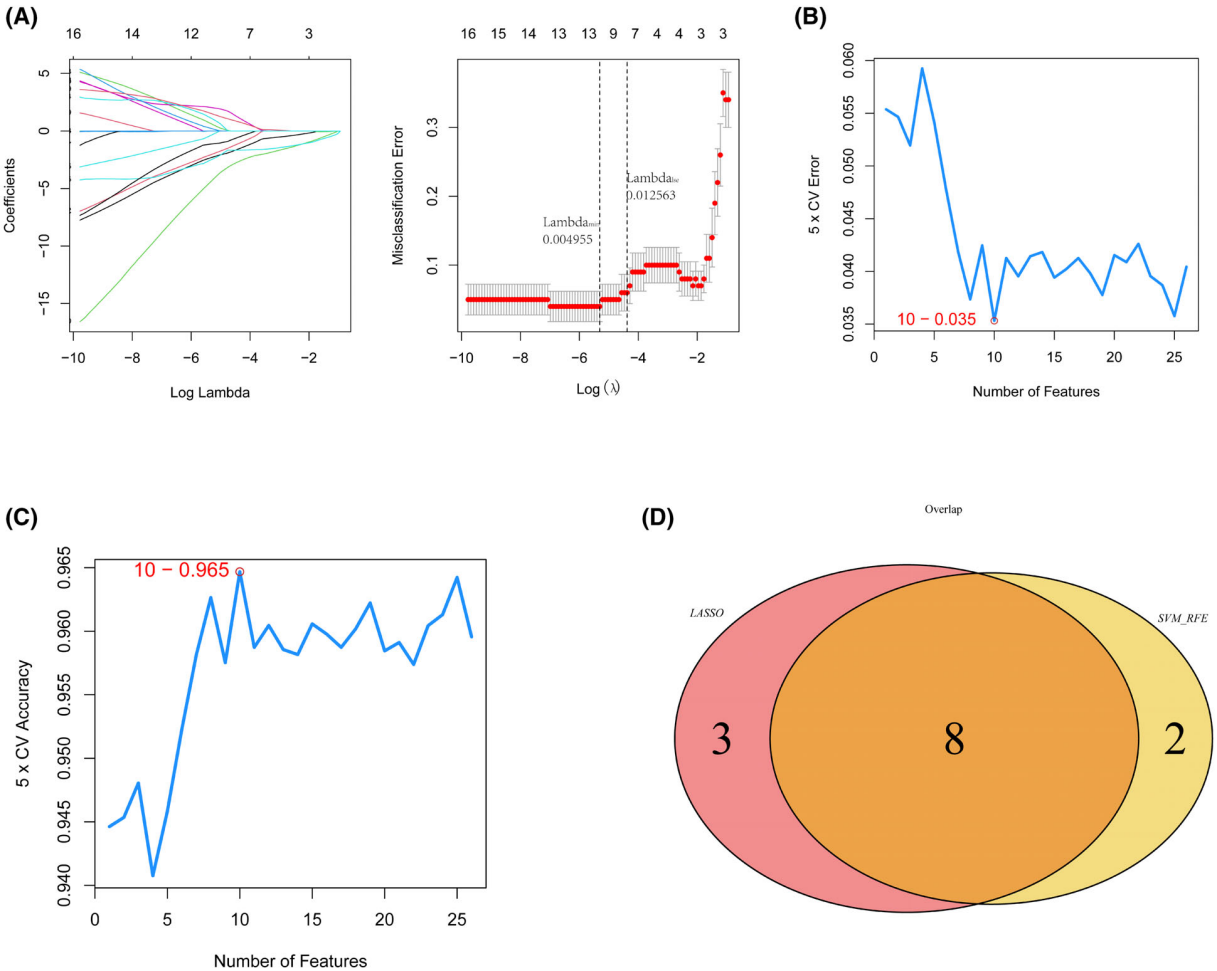
Diagnostic markers of CAD. (A) LASSO regression analysis was conducted to screen the characteristic genes [abscissa deviance indicates the proportion of residual error explained by the model, indicating the correlation between the number of characteristic genes and the proportion of residual error explained (dev), ordinate is the coefficient of the gene (left); abscissa is log (λ), ordinate represents the error of cross‐validation (right)]. (B, C) SVM feature number with error rate and precision rate. (D) The intersection of LASSO feature genes with SVM feature genes.

To further select the optimal candidate diagnostic genes with significant eigenvalues distinguishing CAD patients from normal patients, we used the LASSO algorithm to identify 11 genes (Fig. 4A) and the SVM‐RFE algorithm to identify 10 genes (Fig. 4B,C). After the intersection of the genes selected by LASSO and SVM‐RFE algorithms, eight genes (*CCNDBP1*, *CDC42SE1*, *ERCC5*, *HES6*, *PCSK1N*, *PTGDS*, *RAB2A* and *RORA*) were identified by the mentioned two algorithms simultaneously (Fig. 4D) and were regarded as the optimal candidate features for classification CAD diagnosis.

### Evaluation and validation of diagnostic value of optimal candidate features

**Fig. 5:**
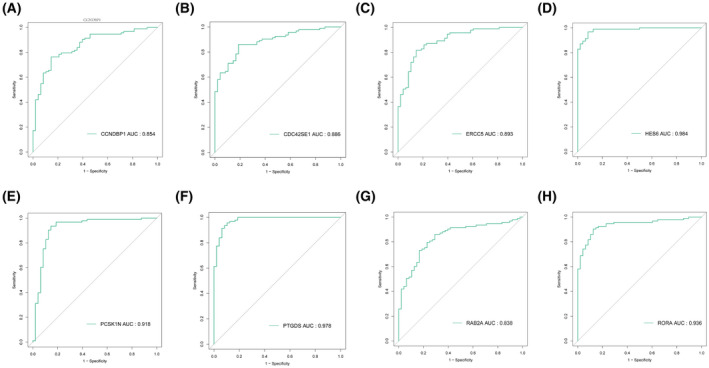
ROC curve validation of candidate diagnostic genes (AUC refers to the surface area under the ROC curve). (A) *CCNDBP1*, (B) *CDC42SE1*, (C) *ERCC5*, (D) *HES6*, (E) *PCSK1N*, (F) *PTGDS*, (G) *RAB2A* and (H) *RORA*.

To determine which candidate features are diagnostic for CAD patients, ROC analysis was conducted to investigate the sensitivity and specificity of the mentioned genes for CAD diagnosis. As revealed from the results, the mentioned eight candidate features may have optimal diagnostic value with respect to distinguishing CAD patients from healthy people: *CCNDBP1* (AUC = 0.854), *CDC42SE1* (AUC = 0.886), *ERCC5* (AUC = 0.893), *HES6* (AUC = 0.984), *PCSK1N* (AUC = 0.918), *PTGDS* (AUC = 0.978), *RAB2A* (AUC = 0.838) and *RORA* (AUC = 0.936) (Fig. 5).

**Fig. 6:**
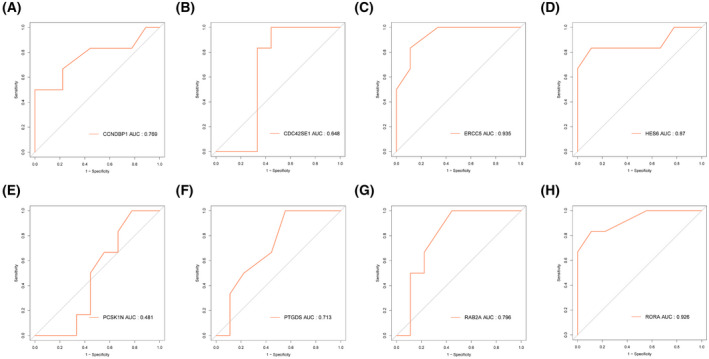
ROC curve validation of candidate diagnostic genes in validation set GSE23561. (A) *CCNDBP1*, (B) *CDC42SE1*, (C) *ERCC5*, (D) *HES6*, (E) *PCSK1N*, (F) *PTGDS*, (G) *RAB2A* and (H) *RORA*.

Furthermore, the mentioned candidate diagnostic features in the validation set (GSE23561 data set) were validated. As a result, the AUCs of the mentioned eight candidate diagnostic genes consisted of *CCNDBP1* (AUC = 0.769), *CDC42SE*1 (AUC = 0.648), *ERCC5* (AUC = 0.935), *HES6* (AUC = 0.87), *PCSK1N* (AUC = 0.481), *PTGDS* (AUC = 0.713), *RAB2A* (AUC = 0.796) and *RORA* (AUC = 0.926) (Fig. 6). On the whole, as suggested from the mentioned results, the expression of diagnostic markers *ERCC5*, *HES6* and *RORA* (AUC > 0.8) is correlated with the progression of CAD and they may be used as biomarkers to assess the pathogenesis of CAD and validate the effectiveness of CAD treatment.

### Functional enrichment analysis of diagnostic genes

**Fig. 7:**
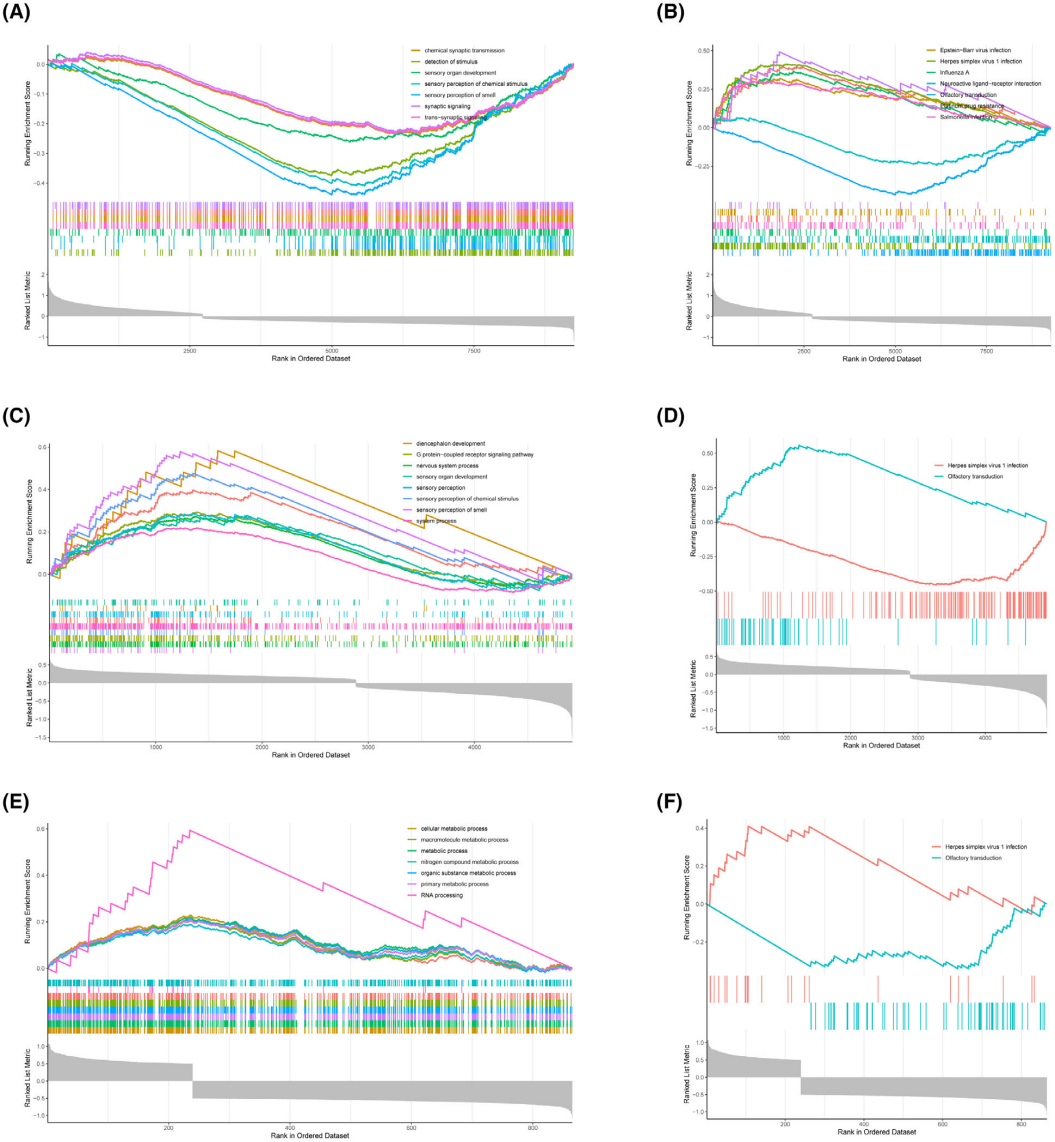
Functional enrichment of diagnostic genes. (A, B) Single‐gene GSEA of *ERCC5* enriched for Gene Ontology‐Biological Process (GO‐BP) and Kyoto Encyclopedia of Genes and Genomes (KEGG) results. (C, D) Single‐gene GSEA of *HES6* enriched for GO‐BP and KEGG results. (E, F) Single‐gene GSEA enrichment of GO‐BP and KEGG results for *RORA*.

**Fig. 8:**
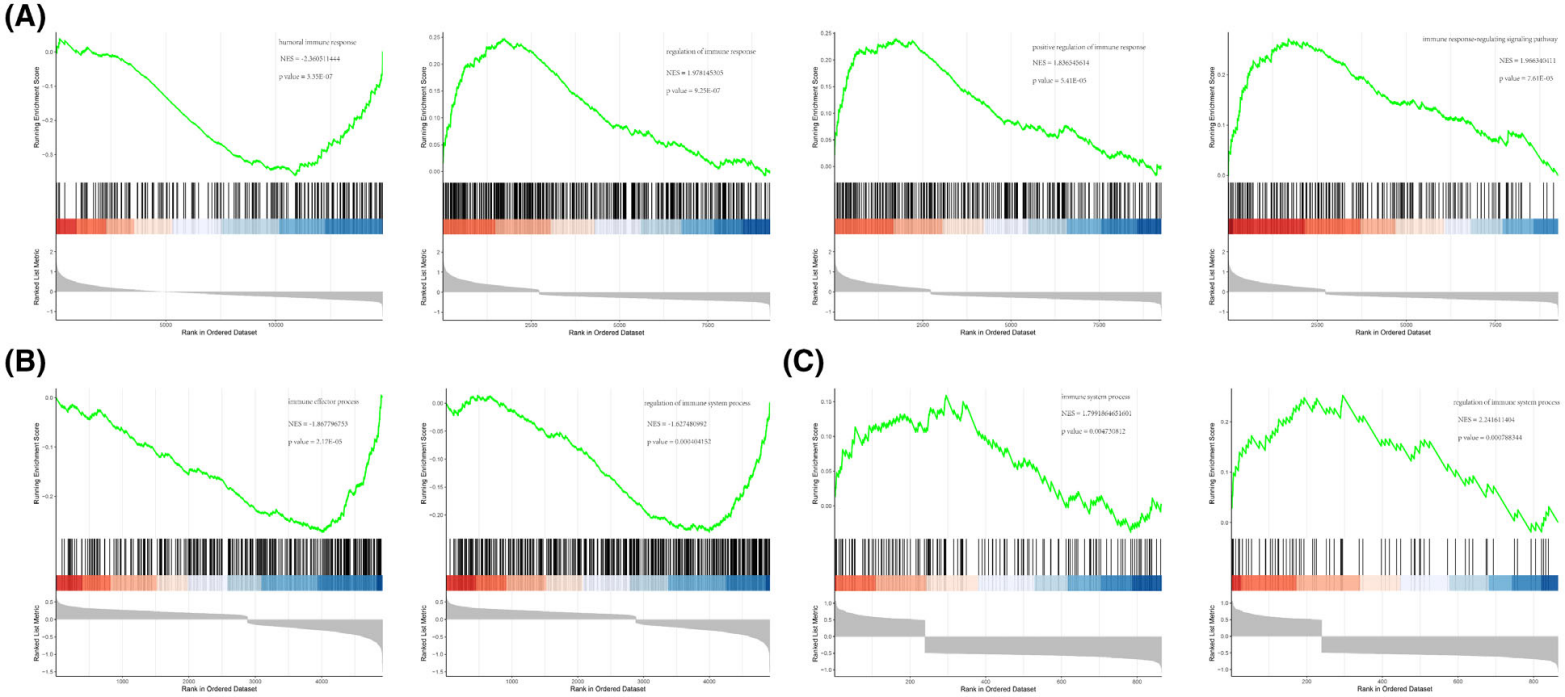
Immune‐correlated pathways of diagnostic genes. (A) Immune‐correlated pathways of single‐gene GSEA of *ERCC5*. (B) Immune‐correlated pathways by single‐gene GSEA of *HES6*. (C) Immune‐correlated pathways of single‐gene GSEA for *RORA*.

**Fig. 9:**
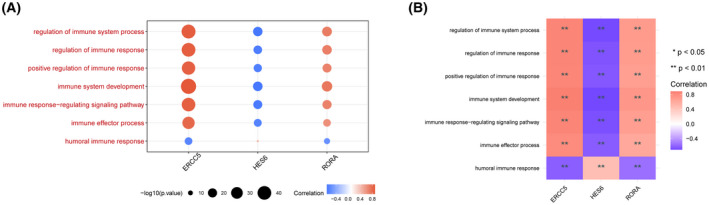
Correlations analysis among immune‐related pathways and three diagnostic genes. (A) Bubble plot. (B) Heatmap. Blue indicates positive correlations and red represents negative correlations; a larger circle size reflects a smaller *P* value, **P < 0.01.

Through gene set enrichment analysis, we found a complete list of gene sets enriched in samples with *ERCC5* (Fig. 7A, B), *HES6* (Fig. 7C,D) and *RORA* (Fig. 7E, F) expression. We then selected the set of genes correlated with immunity from the complete list for further analysis. Humoral immune response was significantly enriched in *ERCC5* low expression samples, whereas three gene sets were significantly enriched in *ERCC5* high expression samples, including regulation of immune response, positive regulation of immune response and immune response‐regulating signaling pathway (Fig. 8A). In addition, immune effect or process was significantly enriched in samples with high *HES6* or *RORA* expression, whereas regulation of immune system process was significantly enriched in samples with low *HES6* or *RORA* expression (Fig. 8B, C). Furthermore, we analyzed the correlations among immune‐correlated pathways and three diagnostic genes. The results suggested that *ERCC5* and *RORA* were significantly positively correlated with regulation of immune response, positive regulation of immune response, immune response‐regulating signaling pathway, immune effector process, immune system development and regulation of immune system process, but negatively correlated with humoral immune response, whereas *HES6* demonstrated the opposite (Fig. 9).

### Expression and validation of diagnostic genes

**Fig. 10:**
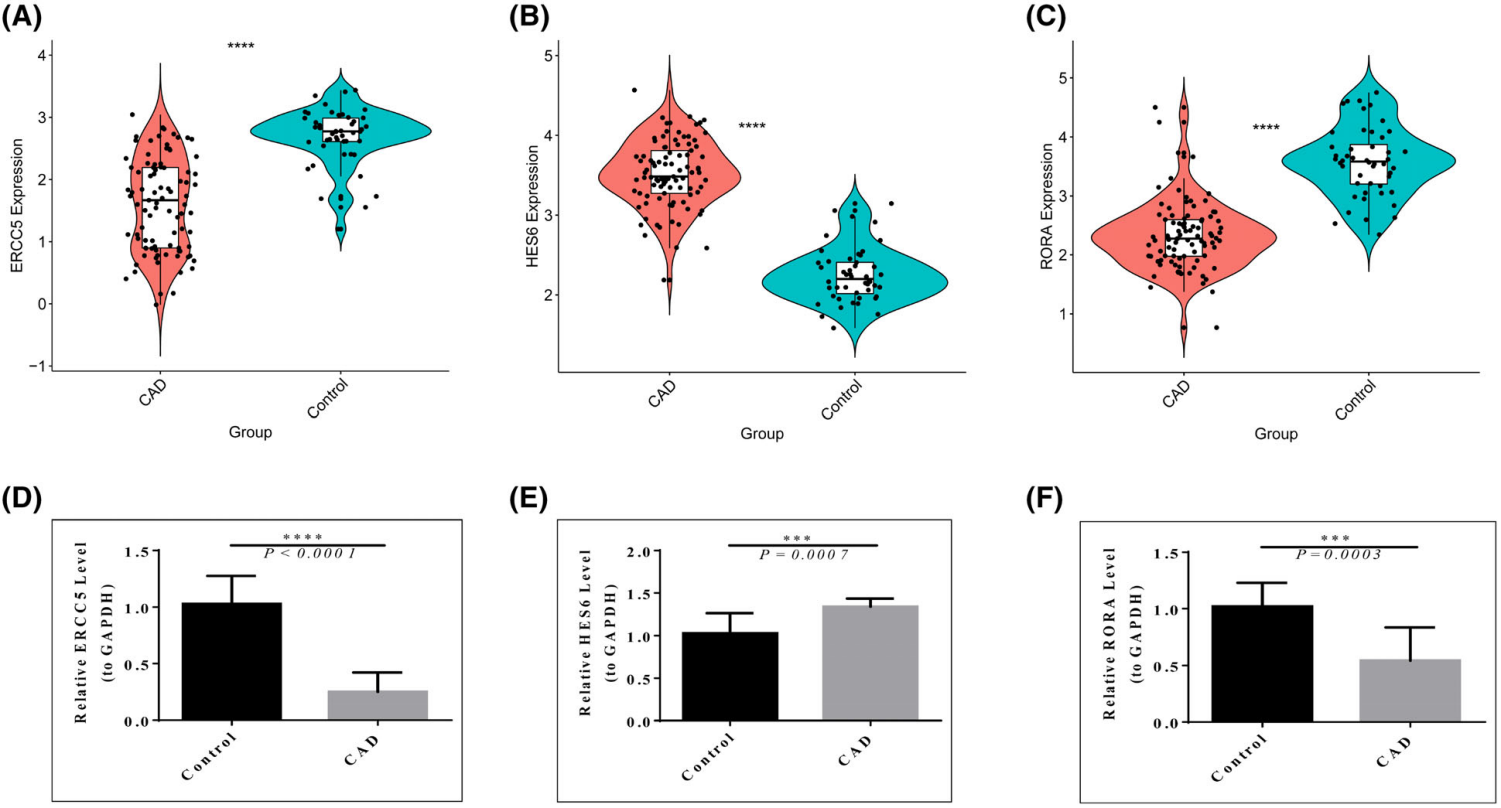
Diagnostic gene expression assays. (A–C) The expression of diagnostic genes between CAD and control samples in the GEO data. (D–F) Expression of diagnostic genes between clinical CAD and control samples. Error bars indicate the SEM (*n* = 10). There was statistical significance in the intergroup rank‐sum test, ***P < 0.001, ****P < 0.0001.

The expression of diagnostic genes (i.e. *ERCC5*, *HES6* and *RORA*) in CAD and normal samples was found in the GEO data set. As revealed from the results, the expression of *ERCC5* and *RORA* was significantly down‐regulated in CAD compared to those of the control (Fig. 10A, C), whereas *HES6* expression was up‐regulated in the CAD samples (Fig. 10B). The above results complied with the detected results of clinical blood samples, such that the expression of *ERCC5* and *RORA* was significantly down‐regulated in CAD patients compared to those of the normal group in blood samples (Fig. 10D,F), whereas *HES6* expression was up‐regulated (Fig. 10E). These results suggested that the diagnostic markers (i.e. *ERCC5*, *HES6* and *RORA*) are correlated with the progression of CAD and may serve as the biomarkers for the effectiveness of CAD treatment.

## Discussion

The main cause of CAD has been reported as atherosclerosis, a multifactorial disease characterized by intimal injury [15]. The identification of early biomarkers of CAD is part of risk prediction. Accordingly, molecular markers and the underlying molecular mechanisms of CAD development should be investigated in depth for the diagnosis and early treatment of CAD.

We first analyzed DEGs in peripheral blood of CAD patients and controls, taking an intersection with genes selected in WGCNA, and 26 vital genes were screened. Through the LASSO and SVM‐RFE algorithms, eight candidate genes were selected to explore their diagnostic value as CAD biomarkers. According to the data of this study, *ERCC5*, *HES6* and *RORA* (AUC > 0.8) can serve as novel biomarkers for the diagnosis of CAD.

The mentioned diagnostic markers were reported to be correlated with the development of various human diseases. *ERCC5*, pertaining to the FEN1/XPG endonuclease family, is ectopically expressed in gastric cancer, breast cancer, scaly cell carcinoma and liver cancer [16]. *ERCC5* single nucleotide polymorphisms are capable of affecting DNA repair capacity and thus interfering with cancer susceptibility [17]. An up‐regulated expression of *HES6*, a helix–loop–helix transcriptional repressor, could significantly increase the invasive phenotype and decrease survival in prostate cancer and glioma [18, 19]. *HES6* was found to be up‐regulated only at the transcriptional level in a xenograft model of metastatic colorectal cancer [20]. As revealed from the single‐cell RNA sequencing results, *HES6* could stimulate the metastasis in primary uveal melanoma [21]. Moreover, *HES6* was suggested to inhibit HES1 function and promote neural stem cell differentiation by binding to HES1 [22]. It is noteworthy that *RORA* is expressed in numerous brain tissues (e.g. the cerebellum, pineal gland and hippocampus) and it was demonstrated to be correlated with autism and cerebral ataxia [23]. Furthermore, *RORA* activation increased the secretion of miR‐122 to plasma in the liver, thereby up‐regulating miR‐122 levels in distal tissues (e.g. muscle and myocardium) [24]. However, to the best of our knowledge, we report initially that *ERCC5*, *HES6* and *RORA* can serve as diagnostic markers for CAD.

To examine the effect exerted by diagnostic markers in the development of CAD, we investigated the biological processes involved in *ERCC5*, *HES6* and *RORA*. As revealed from the results, *ERCC5*, *HES6* and *RORA* were correlated with humoral immune response, the regulation of immune response, positive regulation of immune response, immune response‐regulating signaling pathway, immune effect or process and regulation of immune system process immune‐correlated biological processes. There is increasing evidence that the immune response is correlated with the pathogenesis of CAD [25, 26, 27]. CAD is recognized as a complex chronic inflammatory process triggered by cardiovascular risk factors, thereby causing endothelial dysfunction and inflammatory cell infiltration in the arterial wall. Moreover, Toll‐like receptors are critical to the pathogenesis of CAD interms of being the potent inflammatory cytokines [28].

However, some limitations were reported in the present study. For example, other vital genes selected in WGCNA may be excluded and the sample size of CAD patients (*n* = 10) for qRT‐PCR analysis was small.

With the assistance of bioinformatics methods, the present study reported *ERCC5*, *HES6* and *RORA* as biomarkers for CAD diagnosis based on WGCNA initially, in addition to their molecular mechanisms in the development of CAD. Furthermore, the mentioned three diagnostic markers were experimentally validated by performing PCR experiments. These genes and associated immune pathways may play an important role in the pathogenesis of CAD. Nevertheless, further experiments are required to explore the specific mechanism of action. Additionally, it is necessary to perform further investigations into the drugs interacting with these genes/gene products, which is of certain clinical significance for the accurate treatment of CAD in the future.

## Conclusions

In the present study, *ERCC5*, *HES6* and *RORA* were identified as the biomarkers distinguishing CAD from the normal population based on bioinformatics methods. In addition, their possible regulatory mechanisms were explored via immune pathways, thereby presenting a novel direction for the early diagnosis and treatment of CAD.

## Conflicts of interest

The authors declare that they have no conflicts of interest.

## Author contributions

ZB and YL designed the research experiments. ZB and YL performed the experiments and acquired data. ZB, YL and LT conceived the study and supervised the project. All authors read and approved the final manuscript submitted for publication.

## Supporting information


**Fig. S1.** (A) Hierarchical cluster trees and (B) heatmap of clinical traits (branches represent samples and ordinate represents the height of hierarchical clusters. The branch refers to a red clinical trait representing sample pertaining to such a trait).

**Table S1.** Characteristics of CAD patients and normal individuals.
**Table S2.** Genes differentially expressed between CAD and normal samples.

## Data Availability

Publicly available datasets were analyzed in this study. All of the raw data used in this study are derived from the public GEO data portal (https://www.ncbi.nlm.nih.gov/geo/; Accession numbers: GSE113079, GSE23561).
