# Supplementary figures and images for: ERCC5 , HES6 and RORA are potential diagnostic markers of coronary artery disease
Source: FEBS Open Bio. 2022 Aug 7;12(10):1814–27. doi: 10.1002/2211-5463.13469 (PMC9527589; doi:10.1002/2211-5463.13469)

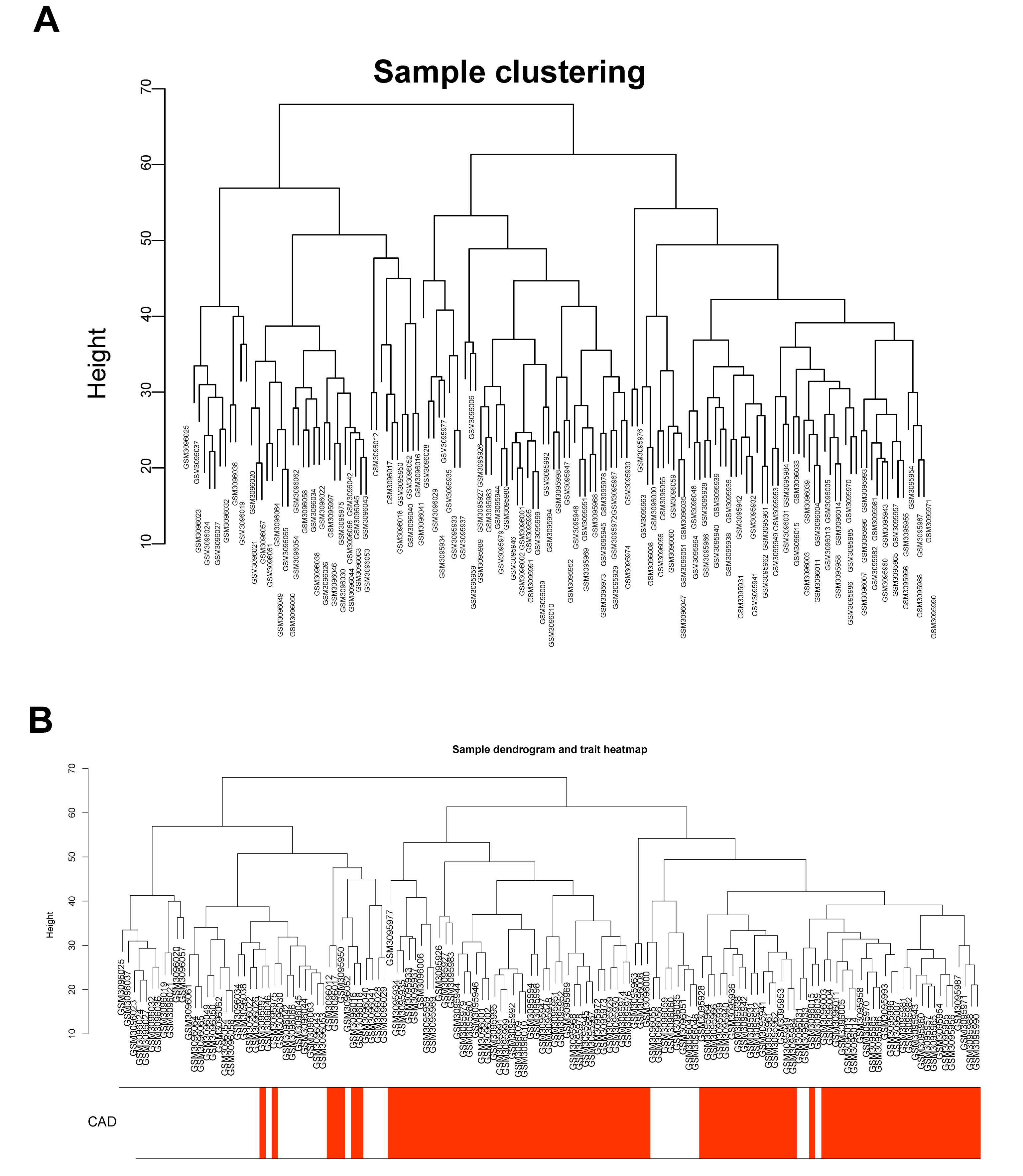

Supplement: Supplementary file 1 — Fig. S1. (A) Hierarchical cluster trees and (B) heatmap of clinical traits (branches represent samples and ordinate represents the height of hierarchical clusters. The branch refers to a red clinical trait representing sample pertaining to such a trait). [file FEB4-12-1814-s001.png]
