# Supplementary material for: ERCC5 , HES6 and RORA are potential diagnostic markers of coronary artery disease
Source: FEBS Open Bio. 2022 Aug 7;12(10):1814–27. doi: 10.1002/2211-5463.13469 (PMC9527589; doi:10.1002/2211-5463.13469)
Supplement: Supplementary file 2 — Table S1. Characteristics of CAD patients and normal individuals. Table S2. Genes differentially expressed between CAD and normal samples. [file FEB4-12-1814-s002.docx]

Supplementary Data

# Supplementary Tables

Supplementary Table 1. Characteristics of CAD patients and normal persons.

| No. | Gender | Age (y) |
| --- | --- | --- |
| CAD Patients | | |
| P1 | Female | 72 |
| P2 | Male | 58 |
| P3 | Male | 66 |
| P4 | Male | 55 |
| P5 | Female | 79 |
| P6 | Male | 43 |
| P7 | Female | 62 |
| P8 | Male | 73 |
| P9 | Female | 67 |
| P10 | Male | 54 |
| Normal Persons | | |
| N1 | Female | 73 |
| N2 | Male | 45 |
| N3 | Female | 78 |
| N4 | Male | 50 |
| N5 | Male | 57 |
| N6 | Male | 68 |
| N7 | Male | 55 |
| N8 | Female | 60 |
| N9 | Male | 75 |
| N10 | Female | 70 |
| N8 | Male | 80 |

Supplementary Table 2 Genes differentially expressed between CAD and normal samples.

|  | logFC | AveExpr | t | P.Value | adj.P.Val | B |
| --- | --- | --- | --- | --- | --- | --- |
| DUX4 | 1.276905972 | -0.934978767 | 19.59536933 | 2.29E-42 | 3.41E-38 | 85.8111208 |
| KIF17 | 1.93029523 | -1.833432541 | 18.65251237 | 3.72E-40 | 1.38E-36 | 80.78298164 |
| SHANK1 | 1.669136328 | 1.09347355 | 18.52793826 | 7.34E-40 | 2.18E-36 | 80.11015496 |
| POU3F3 | 1.060130998 | 0.078478512 | 18.11187818 | 7.22E-39 | 1.79E-35 | 77.84884693 |
| PTGDS | 1.003831167 | 2.508135436 | 18.05195377 | 1.01E-38 | 2.14E-35 | 77.52137177 |
| GCLM | 1.256857424 | 4.08694496 | 17.99892768 | 1.35E-38 | 2.49E-35 | 77.23122273 |
| HES6 | 1.268366539 | 3.085934068 | 17.97896747 | 1.51E-38 | 2.49E-35 | 77.1219139 |
| SHROOM4 | 1.12892033 | 0.113832991 | 17.95797389 | 1.69E-38 | 2.52E-35 | 77.00689276 |
| OPN4 | 1.795965845 | -1.450169845 | 17.585659 | 1.34E-37 | 1.54E-34 | 74.95802387 |
| LCN8 | 1.249688792 | -0.005306461 | 17.09929123 | 2.06E-36 | 2.04E-33 | 72.256229 |
| WFDC2 | 1.500958776 | -0.818696754 | 16.83856224 | 8.99E-36 | 8.36E-33 | 70.7963382 |
| KLKB1 | 1.248021319 | 0.393127954 | 16.80275091 | 1.10E-35 | 9.64E-33 | 70.59520388 |
| PPP1R3F | 1.073269253 | 0.271266188 | 16.75844478 | 1.42E-35 | 1.17E-32 | 70.34615414 |
| PRMT5 | 1.088880393 | 1.511844587 | 16.70521633 | 1.92E-35 | 1.50E-32 | 70.04665301 |
| PKMYT1 | 1.125338836 | 0.676024699 | 16.61424936 | 3.22E-35 | 2.34E-32 | 69.53405921 |
| YY2 | 1.001666857 | 2.000785222 | 16.60962899 | 3.30E-35 | 2.34E-32 | 69.50799855 |
| MYBPC3 | 1.093359725 | -1.847385275 | 16.39347103 | 1.13E-34 | 7.34E-32 | 68.28609772 |
| PHF21A | 1.114567483 | 3.273166715 | 16.34102637 | 1.53E-34 | 9.49E-32 | 67.98885104 |
| TPSD1 | 1.180244788 | -1.980241299 | 16.31742215 | 1.75E-34 | 1.04E-31 | 67.85496739 |
| OR10AD1 | 1.102464545 | 0.145175036 | 16.28460952 | 2.12E-34 | 1.21E-31 | 67.66875136 |
| TNXB | 1.001473998 | 2.819315167 | 16.27098223 | 2.29E-34 | 1.26E-31 | 67.59137986 |
| DEFB1 | 1.042554447 | 0.798995279 | 16.25570166 | 2.50E-34 | 1.33E-31 | 67.50459733 |
| GPR32 | 1.071064657 | -0.217156465 | 16.23005596 | 2.89E-34 | 1.48E-31 | 67.3588909 |
| GNG2 | -1.088222733 | 2.251382832 | -16.1583195 | 4.37E-34 | 2.04E-31 | 66.95093804 |
| TRPM5 | 1.770094478 | -2.260785361 | 16.15716676 | 4.40E-34 | 2.04E-31 | 66.94437803 |
| BHLHA9 | 1.132665576 | 0.047238065 | 16.14322187 | 4.76E-34 | 2.13E-31 | 66.8650092 |
| MUC3A | 1.441706392 | -1.825584202 | 16.1391624 | 4.88E-34 | 2.13E-31 | 66.84190029 |
| NPB | 1.055003528 | -2.035284208 | 16.10928795 | 5.79E-34 | 2.46E-31 | 66.67178272 |
| BEST3 | 1.272730298 | -1.063024095 | 16.09085602 | 6.44E-34 | 2.66E-31 | 66.56677558 |
| SLC22A31 | 1.005369618 | 1.45921305 | 16.07272682 | 7.14E-34 | 2.87E-31 | 66.46345742 |
| OR4C3 | 1.628095659 | -0.790496269 | 16.00684329 | 1.04E-33 | 3.98E-31 | 66.08769105 |
| CCDC8 | 1.344625463 | -1.390156352 | 16.00569119 | 1.05E-33 | 3.98E-31 | 66.08111589 |
| BIRC7 | 1.984138947 | -2.56047815 | 16.00271759 | 1.07E-33 | 3.98E-31 | 66.06414469 |
| HBM | 1.124003721 | 0.783720065 | 15.98701929 | 1.17E-33 | 4.25E-31 | 65.97453424 |
| ZAN | 1.366550827 | 0.25888176 | 15.97701398 | 1.24E-33 | 4.26E-31 | 65.91740733 |
| IL17REL | 1.372826241 | -0.224901641 | 15.97411899 | 1.26E-33 | 4.26E-31 | 65.90087596 |
| SPDYE4 | 1.127302079 | -2.211467236 | 15.83635387 | 2.79E-33 | 8.84E-31 | 65.1131666 |
| NUPR1 | 1.733215102 | -2.861221267 | 15.77129892 | 4.07E-33 | 1.24E-30 | 64.74050656 |
| DCUN1D1 | 1.004992483 | 0.354027498 | 15.75951279 | 4.35E-33 | 1.30E-30 | 64.672944 |
| EGFL7 | 1.23995844 | -2.149442531 | 15.5892722 | 1.17E-32 | 3.34E-30 | 63.69547114 |
| PALM3 | 1.017604945 | -0.000494143 | 15.58517637 | 1.20E-32 | 3.36E-30 | 63.67191774 |
| KCNK9 | 1.215797612 | -1.960401061 | 15.57038822 | 1.30E-32 | 3.59E-30 | 63.58686316 |
| PAX8 | 1.448894007 | -2.747386 | 15.54586912 | 1.50E-32 | 4.07E-30 | 63.44579225 |
| HSD3B1 | 1.233123379 | -1.392231369 | 15.51618142 | 1.79E-32 | 4.74E-30 | 63.27490297 |
| OR1J1 | 1.240397822 | -2.270738808 | 15.5088154 | 1.86E-32 | 4.87E-30 | 63.23248882 |
| CNTN4 | 1.007321422 | -0.883373494 | 15.46695931 | 2.38E-32 | 6.10E-30 | 62.99137542 |
| CDRT15L2 | 1.433893962 | -0.941503781 | 15.452549 | 2.58E-32 | 6.52E-30 | 62.90832415 |
| ADM | 1.040057301 | 2.642670328 | 15.28749062 | 6.76E-32 | 1.60E-29 | 61.95558539 |
| SLC4A11 | 1.010303173 | -1.154621966 | 15.19858022 | 1.14E-31 | 2.52E-29 | 61.44129087 |
| LCE1A | 1.07168524 | 0.260694802 | 15.17549861 | 1.30E-31 | 2.85E-29 | 61.30765423 |
| FAM169A | -1.790242692 | -1.650196413 | -15.10977576 | 1.91E-31 | 4.00E-29 | 60.92686064 |
| PCDHB8 | 1.177169942 | -2.435342775 | 15.06011631 | 2.55E-31 | 5.28E-29 | 60.63886953 |
| GRIN3B | -1.134376635 | -0.446248308 | -15.05118825 | 2.69E-31 | 5.41E-29 | 60.58706856 |
| TSPO2 | 1.113068142 | -0.288070305 | 15.03569682 | 2.95E-31 | 5.84E-29 | 60.49716926 |
| NMNAT2 | 1.866523188 | -2.474408192 | 15.02701366 | 3.10E-31 | 6.07E-29 | 60.44676972 |
| PRAMEF12 | 1.166135777 | -1.284934133 | 15.01894319 | 3.25E-31 | 6.28E-29 | 60.39992029 |
| FAIM2 | 1.387088282 | -0.808947306 | 14.99803119 | 3.67E-31 | 7.01E-29 | 60.27849729 |
| CPEB1 | 1.644318305 | -3.270500975 | 14.98236553 | 4.03E-31 | 7.58E-29 | 60.18751034 |
| ZNF492 | 1.415228195 | -1.785040147 | 14.97381284 | 4.23E-31 | 7.87E-29 | 60.13782642 |
| SPACA5 | 1.176181879 | -1.553204817 | 14.96008177 | 4.59E-31 | 8.43E-29 | 60.0580466 |
| FTMT | 1.803873283 | -0.959906663 | 14.91047287 | 6.14E-31 | 1.10E-28 | 59.76966829 |
| BCAM | 1.013125633 | -0.581176782 | 14.89982935 | 6.53E-31 | 1.16E-28 | 59.70776825 |
| SLC5A11 | 1.007054468 | -0.880210219 | 14.88386042 | 7.18E-31 | 1.24E-28 | 59.61487792 |
| PPIAL4G | 1.284915865 | 3.837657225 | 14.87481547 | 7.57E-31 | 1.29E-28 | 59.56225388 |
| KRT32 | 1.43901152 | -0.578953871 | 14.85083491 | 8.71E-31 | 1.47E-28 | 59.42269825 |
| TRIM47 | 1.390785877 | -1.678945225 | 14.75545656 | 1.53E-30 | 2.47E-28 | 58.86713835 |
| TM4SF5 | 1.617365888 | -2.209790804 | 14.68945008 | 2.25E-30 | 3.52E-28 | 58.48219969 |
| KITLG | 1.435174602 | -1.909305628 | 14.66946922 | 2.53E-30 | 3.92E-28 | 58.36560085 |
| LMAN1L | 1.113898558 | -2.137945419 | 14.66030154 | 2.67E-30 | 4.06E-28 | 58.31209119 |
| COL23A1 | 1.46851262 | -3.091949876 | 14.6509184 | 2.82E-30 | 4.24E-28 | 58.25731657 |
| IGFALS | 1.090998264 | -1.795622832 | 14.63635066 | 3.08E-30 | 4.58E-28 | 58.17226171 |
| ODF4 | 1.202731724 | -1.934197117 | 14.60822899 | 3.63E-30 | 5.30E-28 | 58.00802042 |
| CFC1 | 1.006828848 | -0.691144875 | 14.55403608 | 5.00E-30 | 6.95E-28 | 57.69132611 |
| CORT | 1.069699598 | -1.413334696 | 14.53110877 | 5.72E-30 | 7.88E-28 | 57.5572692 |
| PLEKHN1 | 1.003595171 | 1.403546312 | 14.5212842 | 6.06E-30 | 8.13E-28 | 57.49981123 |
| PIH1D2 | 1.504389388 | -2.289441033 | 14.38334703 | 1.37E-29 | 1.78E-27 | 56.69227104 |
| CDR2L | 1.109889754 | -1.938176968 | 14.38255771 | 1.38E-29 | 1.78E-27 | 56.68764567 |
| SLC4A1 | 1.070905736 | -1.281078767 | 14.38219578 | 1.38E-29 | 1.78E-27 | 56.68552474 |
| TBC1D26 | 1.113958044 | -1.096949965 | 14.36123175 | 1.56E-29 | 2.00E-27 | 56.56265729 |
| RNF125 | -1.306399757 | 0.283707093 | -14.34015234 | 1.77E-29 | 2.25E-27 | 56.43907866 |
| CEP120 | -1.055346669 | -1.515693532 | -14.25420259 | 2.94E-29 | 3.68E-27 | 55.93483717 |
| PLA2R1 | 1.670650799 | -2.567764841 | 14.24842626 | 3.04E-29 | 3.77E-27 | 55.90092873 |
| RNF207 | 1.17944538 | -2.39403379 | 14.24423404 | 3.12E-29 | 3.84E-27 | 55.87631784 |
| AQP8 | 1.083804424 | -2.256007381 | 14.13636047 | 5.91E-29 | 6.98E-27 | 55.24257656 |
| GRIK5 | 1.40636245 | -1.137918531 | 14.08381407 | 8.07E-29 | 9.39E-27 | 54.93356118 |
| CEND1 | 1.104487035 | 0.383469426 | 14.04993971 | 9.87E-29 | 1.12E-26 | 54.73424599 |
| CX3CL1 | 1.103451918 | -1.533771316 | 13.99228778 | 1.39E-28 | 1.52E-26 | 54.39483563 |
| CLEC4G | 1.142182716 | -0.062915509 | 13.94902275 | 1.80E-28 | 1.92E-26 | 54.1399703 |
| TNNI2 | 1.017691139 | 1.754557941 | 13.7828717 | 4.83E-28 | 4.82E-26 | 53.16001952 |
| PTGER1 | 1.261770628 | -2.494678262 | 13.73684704 | 6.36E-28 | 6.18E-26 | 52.88824495 |
| F2R | -1.644269971 | -3.558319312 | -13.72715483 | 6.73E-28 | 6.51E-26 | 52.83099529 |
| PYDC2 | 1.679579479 | -3.018818242 | 13.70629662 | 7.62E-28 | 7.32E-26 | 52.70777035 |
| GPN1 | -1.138970953 | -0.345710482 | -13.45895281 | 3.34E-27 | 2.97E-25 | 51.24449729 |
| TOPORS | -1.149078272 | 0.351663335 | -13.43325848 | 3.89E-27 | 3.42E-25 | 51.09228575 |
| IL18RAP | -1.441303633 | 1.011968228 | -13.39633497 | 4.85E-27 | 4.20E-25 | 50.87348893 |
| AVPR1B | 1.72547893 | -2.563629985 | 13.38343221 | 5.24E-27 | 4.51E-25 | 50.79701363 |
| MYBL1 | -1.203241825 | 0.901925063 | -13.37702035 | 5.44E-27 | 4.66E-25 | 50.75900691 |
| NOG | 1.539884418 | -0.076595225 | 13.36825556 | 5.74E-27 | 4.88E-25 | 50.7070494 |
| TRIM10 | 1.227124216 | -2.556414746 | 13.30924868 | 8.17E-27 | 6.83E-25 | 50.35715096 |
| ARL4C | -1.040676898 | 2.974287708 | -13.28539138 | 9.42E-27 | 7.83E-25 | 50.21563028 |
| KRTAP10-5 | 1.142226137 | -2.050989643 | 13.2765204 | 9.93E-27 | 8.16E-25 | 50.16300039 |
| CSNK1A1 | -1.974052219 | -1.263071555 | -13.26407426 | 1.07E-26 | 8.75E-25 | 50.08915306 |
| GPR31 | 1.240396088 | -2.749559521 | 13.24830841 | 1.18E-26 | 9.56E-25 | 49.99559739 |
| IDI1 | -1.332572547 | 2.081785777 | -13.21381623 | 1.45E-26 | 1.15E-24 | 49.79087487 |
| SEMA5B | 1.336902595 | -2.001505336 | 13.15929814 | 2.00E-26 | 1.57E-24 | 49.46717291 |
| FOXJ1 | 1.059039262 | -0.459006392 | 13.15736562 | 2.03E-26 | 1.58E-24 | 49.45569591 |
| OR10H1 | 1.074596433 | -2.141962474 | 13.1434787 | 2.20E-26 | 1.70E-24 | 49.37321799 |
| BRF2 | -1.107567684 | -3.575258572 | -13.14124486 | 2.23E-26 | 1.71E-24 | 49.35994984 |
| RCN2 | -1.589119056 | -1.595084493 | -13.06106704 | 3.61E-26 | 2.71E-24 | 48.88357025 |
| CEP78 | -1.143673187 | 2.798045079 | -13.04117906 | 4.07E-26 | 3.04E-24 | 48.76536008 |
| C20orf144 | 1.098998923 | -0.32311022 | 13.01464235 | 4.77E-26 | 3.53E-24 | 48.6076042 |
| JOSD1 | -1.058044835 | -0.626893352 | -13.01050921 | 4.89E-26 | 3.60E-24 | 48.58303067 |
| MB | 1.519000342 | -3.493618347 | 12.99037514 | 5.52E-26 | 4.02E-24 | 48.46331345 |
| HOPX | -1.58824662 | 0.012074867 | -12.98901809 | 5.56E-26 | 4.04E-24 | 48.45524384 |
| SYTL2 | -1.419184926 | 0.752563484 | -12.98088708 | 5.84E-26 | 4.22E-24 | 48.40689146 |
| MOS | 1.159107403 | -2.237344993 | 12.97521162 | 6.04E-26 | 4.34E-24 | 48.37313983 |
| CSAG3 | 1.410538229 | -2.978935155 | 12.91096948 | 8.88E-26 | 6.21E-24 | 47.99100257 |
| PIK3R1 | -1.280350439 | -0.250468899 | -12.81251063 | 1.60E-25 | 1.09E-23 | 47.40501588 |
| HRC | 1.177839623 | -1.60484972 | 12.80902967 | 1.64E-25 | 1.11E-23 | 47.38429199 |
| NXT2 | -1.264979586 | -2.269988509 | -12.78225672 | 1.92E-25 | 1.28E-23 | 47.22488456 |
| CATSPER3 | 1.017400395 | -1.809127399 | 12.74806587 | 2.36E-25 | 1.55E-23 | 47.02127377 |
| PDCL | -1.32357972 | -2.87347059 | -12.74709762 | 2.38E-25 | 1.55E-23 | 47.01550711 |
| MUC4 | 1.414643206 | -3.103455377 | 12.67957735 | 3.57E-25 | 2.24E-23 | 46.61329697 |
| SOCS2 | -1.384978734 | -2.87835979 | -12.64583012 | 4.37E-25 | 2.71E-23 | 46.41221365 |
| MYLPF | 1.027572925 | -2.55818278 | 12.62986284 | 4.81E-25 | 2.93E-23 | 46.31706014 |
| IL17B | 1.128277904 | -2.461376143 | 12.60628684 | 5.54E-25 | 3.35E-23 | 46.17655013 |
| PCSK1N | 1.075716046 | 3.418179611 | 12.58907961 | 6.14E-25 | 3.67E-23 | 46.07398687 |
| EXOSC10 | -1.123063783 | -0.518053649 | -12.54533731 | 7.99E-25 | 4.72E-23 | 45.81322421 |
| LY6H | 1.438445746 | -2.70323478 | 12.52500733 | 9.03E-25 | 5.27E-23 | 45.69201245 |
| IL18R1 | -1.652311154 | -1.209052871 | -12.47299704 | 1.23E-24 | 7.06E-23 | 45.38186705 |
| NCAPD3 | -1.084495054 | -2.385183175 | -12.47242195 | 1.24E-24 | 7.06E-23 | 45.37843732 |
| FYN | -1.047315808 | 3.270771186 | -12.47240964 | 1.24E-24 | 7.06E-23 | 45.37836389 |
| C7 | 1.016384042 | 0.232086996 | 12.47189159 | 1.24E-24 | 7.06E-23 | 45.3752743 |
| AKAP5 | -1.683756148 | -0.04093195 | -12.46297685 | 1.31E-24 | 7.42E-23 | 45.32210729 |
| ATP1A4 | 1.474212876 | -0.290195524 | 12.4564525 | 1.36E-24 | 7.69E-23 | 45.28319524 |
| BPIFA1 | 1.154732588 | -1.519224053 | 12.41185305 | 1.78E-24 | 9.87E-23 | 45.01717173 |
| TGFBR3 | -1.256455971 | 2.840174795 | -12.39417002 | 1.98E-24 | 1.09E-22 | 44.91168492 |
| DOHH | 1.039651894 | -0.537528944 | 12.37700014 | 2.20E-24 | 1.21E-22 | 44.80925278 |
| CSMD1 | 1.396985109 | -3.713540606 | 12.34513971 | 2.67E-24 | 1.43E-22 | 44.61916363 |
| MYBPH | 1.12603477 | -1.8896645 | 12.34005057 | 2.75E-24 | 1.47E-22 | 44.5887984 |
| LRRN2 | 1.136739259 | 0.883575302 | 12.31130191 | 3.27E-24 | 1.72E-22 | 44.41725509 |
| TMPRSS11E | 1.23174378 | -2.386182139 | 12.29102638 | 3.69E-24 | 1.92E-22 | 44.29626175 |
| ADRA1A | 1.444613903 | -2.593871146 | 12.28683741 | 3.79E-24 | 1.96E-22 | 44.27126337 |
| REG3A | 1.008415861 | -1.558010851 | 12.28511435 | 3.83E-24 | 1.98E-22 | 44.26098063 |
| KLF3 | -1.330423253 | 0.697657251 | -12.2743704 | 4.08E-24 | 2.10E-22 | 44.19686255 |
| OR2AG1 | 1.255111758 | -2.419480049 | 12.24350215 | 4.91E-24 | 2.48E-22 | 44.01263537 |
| RORA | -1.25366 | 2.748951487 | -12.23457347 | 5.19E-24 | 2.60E-22 | 43.95934458 |
| CYP24A1 | 1.032801156 | -1.278840887 | 12.2067527 | 6.13E-24 | 3.02E-22 | 43.79328876 |
| PDLIM2 | -1.433985979 | -1.110458785 | -12.20668316 | 6.13E-24 | 3.02E-22 | 43.79287365 |
| KRTAP6-1 | 1.144349417 | -2.612234267 | 12.19667373 | 6.52E-24 | 3.19E-22 | 43.7331269 |
| ZCCHC3 | -1.044810094 | -1.583511523 | -12.18641814 | 6.93E-24 | 3.36E-22 | 43.67190935 |
| ACTBL2 | 2.060091607 | 1.523023703 | 12.1749532 | 7.43E-24 | 3.56E-22 | 43.6034713 |
| CBLB | -1.098045631 | 0.385080194 | -12.16696337 | 7.79E-24 | 3.72E-22 | 43.55577628 |
| PKDCC | 1.193967976 | -2.661952188 | 12.10780716 | 1.11E-23 | 5.19E-22 | 43.20262176 |
| USP24 | -1.343499859 | -0.455559999 | -12.08152189 | 1.30E-23 | 6.02E-22 | 43.0456902 |
| RGS22 | 1.310271732 | -2.317126428 | 12.06216708 | 1.46E-23 | 6.65E-22 | 42.93013165 |
| MAGEL2 | 1.063772589 | 0.28222073 | 12.00192277 | 2.11E-23 | 9.35E-22 | 42.570422 |
| SBDS | -1.064603257 | -0.34769966 | -11.99021917 | 2.26E-23 | 9.95E-22 | 42.50053876 |
| GGN | 1.217029195 | -1.16342676 | 11.9884412 | 2.28E-23 | 1.00E-21 | 42.48992228 |
| C1S | 1.070499368 | -1.362399647 | 11.94367013 | 2.99E-23 | 1.28E-21 | 42.22258373 |
| BAG2 | -1.145555021 | -1.977345918 | -11.87392617 | 4.55E-23 | 1.88E-21 | 41.8061136 |
| TMCC2 | 1.037636563 | -0.871514882 | 11.87180572 | 4.61E-23 | 1.90E-21 | 41.79345138 |
| STAG1 | -1.280286988 | -2.852750389 | -11.82607124 | 6.07E-23 | 2.45E-21 | 41.52034966 |
| LHFPL3 | 1.293400156 | -2.233940912 | 11.76077503 | 9.00E-23 | 3.53E-21 | 41.13044249 |
| SYT11 | -1.453842188 | -1.441905768 | -11.7533679 | 9.41E-23 | 3.68E-21 | 41.08621276 |
| DDX23 | -1.082378409 | -1.777159044 | -11.75175384 | 9.51E-23 | 3.70E-21 | 41.07657491 |
| C1orf21 | -1.255906402 | 2.517526747 | -11.73857686 | 1.03E-22 | 3.99E-21 | 40.99789291 |
| KLRB1 | -1.051507432 | 2.371308808 | -11.72055058 | 1.15E-22 | 4.39E-21 | 40.89025638 |
| SMARCAD1 | -1.007928308 | 1.120875831 | -11.71876586 | 1.16E-22 | 4.42E-21 | 40.87959982 |
| POLR3F | -1.212731892 | -1.693403595 | -11.69422358 | 1.34E-22 | 5.04E-21 | 40.73305932 |
| TRIM72 | 1.062377785 | -2.089215434 | 11.65637096 | 1.69E-22 | 6.20E-21 | 40.50705256 |
| RASAL1 | 1.282435119 | -3.377321861 | 11.65310114 | 1.72E-22 | 6.31E-21 | 40.48752999 |
| PARP2 | -1.196622273 | -3.483439867 | -11.6490549 | 1.76E-22 | 6.45E-21 | 40.46337195 |
| JMY | -1.223328211 | -0.149840534 | -11.63802387 | 1.89E-22 | 6.88E-21 | 40.39751205 |
| ITPR3 | -1.283839303 | 2.36263203 | -11.57358617 | 2.78E-22 | 9.97E-21 | 40.01281809 |
| TTC3 | -1.002085274 | 0.607583285 | -11.55254619 | 3.16E-22 | 1.12E-20 | 39.88722012 |
| ASGR1 | 1.185766257 | 0.253059676 | 11.54170653 | 3.37E-22 | 1.19E-20 | 39.82251521 |
| HELT | 1.208067126 | -3.325561007 | 11.51251202 | 4.02E-22 | 1.40E-20 | 39.64825383 |
| S1PR5 | -1.25216751 | 1.12281983 | -11.4984704 | 4.37E-22 | 1.51E-20 | 39.56444443 |
| DACT3 | 1.073857805 | -2.289911316 | 11.44902359 | 5.89E-22 | 2.01E-20 | 39.26934069 |
| JAKMIP2 | -1.297608975 | -0.53160599 | -11.42966589 | 6.62E-22 | 2.24E-20 | 39.15382414 |
| DERL2 | -1.05757244 | -0.368263627 | -11.2985998 | 1.46E-21 | 4.68E-20 | 38.37190252 |
| UHRF1BP1 | -1.048914817 | -0.355180885 | -11.2697425 | 1.73E-21 | 5.49E-20 | 38.19980036 |
| DEFB4A | 1.210071147 | -3.26252469 | 11.26629055 | 1.77E-21 | 5.58E-20 | 38.17921479 |
| SH2D2A | -1.471826603 | 0.975180511 | -11.24554573 | 2.00E-21 | 6.24E-20 | 38.05551074 |
| GPRIN2 | 1.077395216 | -2.353526268 | 11.22791064 | 2.23E-21 | 6.88E-20 | 37.95036013 |
| UNK | -1.066416995 | -0.745585335 | -11.18151279 | 2.95E-21 | 8.93E-20 | 37.67375403 |
| PLAU | -1.033941441 | -0.103866507 | -11.13920816 | 3.80E-21 | 1.15E-19 | 37.42160999 |
| PEX13 | -1.248404182 | 0.014599643 | -11.09964513 | 4.82E-21 | 1.44E-19 | 37.18586162 |
| LYNX1 | 1.363497379 | -3.255241543 | 11.08521957 | 5.26E-21 | 1.56E-19 | 37.0999164 |
| KCNJ16 | 1.243045531 | -3.625990146 | 11.06495075 | 5.94E-21 | 1.75E-19 | 36.97917104 |
| SLC25A36 | -1.285334569 | -1.300173663 | -11.04793714 | 6.58E-21 | 1.93E-19 | 36.87782967 |
| DYRK2 | -1.015744607 | 0.748618097 | -11.04233608 | 6.81E-21 | 1.99E-19 | 36.84446942 |
| ZNF548 | -1.469040812 | -3.626235158 | -11.04172735 | 6.83E-21 | 1.99E-19 | 36.84084391 |
| OR10W1 | 1.046159243 | -2.103588687 | 11.02019153 | 7.78E-21 | 2.24E-19 | 36.71258742 |
| FAM47A | -1.760217133 | -3.452553584 | -11.00822948 | 8.36E-21 | 2.40E-19 | 36.64135559 |
| CRTAC1 | 1.159000884 | -3.740363455 | 10.9971095 | 8.93E-21 | 2.54E-19 | 36.57514331 |
| FXYD1 | 1.006567769 | -2.453932442 | 10.9821078 | 9.78E-21 | 2.76E-19 | 36.48582615 |
| SARS2 | -1.056089473 | -3.051491764 | -10.96297078 | 1.10E-20 | 3.07E-19 | 36.37190193 |
| PDGFRB | -1.612518621 | -3.289093709 | -10.95073693 | 1.18E-20 | 3.28E-19 | 36.29908112 |
| FBXW9 | 1.35127463 | -3.758846493 | 10.94879283 | 1.19E-20 | 3.32E-19 | 36.28750966 |
| KIR2DS4 | -1.82820188 | 1.964758992 | -10.93236477 | 1.32E-20 | 3.63E-19 | 36.18973502 |
| PDGFD | -1.657860277 | -1.77377408 | -10.92483935 | 1.38E-20 | 3.78E-19 | 36.14495017 |
| ABCB1 | -1.008063128 | -0.365534481 | -10.90992921 | 1.51E-20 | 4.09E-19 | 36.05622534 |
| CAND1 | -1.056327131 | 3.126347303 | -10.89558515 | 1.64E-20 | 4.41E-19 | 35.97087875 |
| PITPNB | -1.072841284 | -0.257836906 | -10.88064971 | 1.80E-20 | 4.81E-19 | 35.88202381 |
| OR2J2 | 1.416609338 | -3.137263944 | 10.87277871 | 1.89E-20 | 5.03E-19 | 35.8352014 |
| C22orf31 | -2.097789472 | -1.314511728 | -10.86980406 | 1.92E-20 | 5.10E-19 | 35.81750677 |
| ZBTB11 | -1.223074169 | -1.370762214 | -10.8687577 | 1.93E-20 | 5.12E-19 | 35.81128261 |
| PAQR6 | 1.174275437 | -0.005456508 | 10.86314904 | 2.00E-20 | 5.29E-19 | 35.77792114 |
| SMAD7 | -1.240390735 | -0.457936559 | -10.83342375 | 2.39E-20 | 6.24E-19 | 35.60113485 |
| GNB4 | 1.062057717 | -0.034995634 | 10.82501525 | 2.51E-20 | 6.54E-19 | 35.55113465 |
| B3GNT3 | 1.075026224 | -3.178993062 | 10.81983222 | 2.59E-20 | 6.72E-19 | 35.52031615 |
| PRKAA1 | -1.176017919 | -0.82122288 | -10.79450139 | 3.02E-20 | 7.79E-19 | 35.36971804 |
| PPT2 | -1.003108301 | -3.753776126 | -10.79373166 | 3.03E-20 | 7.81E-19 | 35.36514232 |
| ZNF268 | -1.185310143 | -1.699788328 | -10.77266991 | 3.44E-20 | 8.76E-19 | 35.23995142 |
| LCN1 | 1.008600477 | -1.822671247 | 10.75718741 | 3.77E-20 | 9.52E-19 | 35.1479387 |
| RGPD5 | -1.158382376 | 0.364580499 | -10.74394589 | 4.09E-20 | 1.02E-18 | 35.0692546 |
| ARHGEF33 | -2.781474446 | -3.428818708 | -10.72644095 | 4.54E-20 | 1.13E-18 | 34.96525117 |
| ANTXR1 | 1.460711927 | -3.003846576 | 10.72364517 | 4.61E-20 | 1.14E-18 | 34.94864199 |
| MED18 | 1.492543281 | -1.226592357 | 10.7105822 | 4.99E-20 | 1.22E-18 | 34.87104331 |
| RUFY2 | -1.028482782 | -0.480945789 | -10.69502661 | 5.48E-20 | 1.34E-18 | 34.7786503 |
| WDR47 | -1.178727566 | -4.122962496 | -10.68484855 | 5.82E-20 | 1.41E-18 | 34.71820498 |
| ZFP1 | -1.230235625 | -2.789760428 | -10.68309716 | 5.88E-20 | 1.42E-18 | 34.70780445 |
| KLC3 | 1.272729444 | -3.678752549 | 10.66696197 | 6.48E-20 | 1.55E-18 | 34.61199513 |
| GPX3 | -1.093099979 | 3.530684198 | -10.66006538 | 6.76E-20 | 1.61E-18 | 34.57104857 |
| ZFX | -1.081057279 | -0.721770216 | -10.60223083 | 9.55E-20 | 2.22E-18 | 34.22778633 |
| TOX | -1.292342818 | -1.832342021 | -10.59416705 | 1.00E-19 | 2.31E-18 | 34.17994248 |
| MYF5 | 1.583469061 | -2.65850718 | 10.55257935 | 1.29E-19 | 2.91E-18 | 33.93326171 |
| SMC5 | -1.113004641 | 0.670148243 | -10.5520447 | 1.29E-19 | 2.91E-18 | 33.9300911 |
| CAPN13 | 1.276907842 | -3.195686053 | 10.53059057 | 1.47E-19 | 3.29E-18 | 33.80287963 |
| NCALD | -1.054980787 | -1.125223109 | -10.52546008 | 1.51E-19 | 3.39E-18 | 33.7724632 |
| DTHD1 | -1.261033904 | 0.939704389 | -10.50345094 | 1.73E-19 | 3.82E-18 | 33.6420009 |
| SMURF1 | -1.44278849 | -2.987462753 | -10.49961208 | 1.77E-19 | 3.90E-18 | 33.61924895 |
| CMC1 | -1.374253247 | -0.222257432 | -10.4935704 | 1.83E-19 | 4.03E-18 | 33.58344352 |
| ADD3 | -1.159701761 | 0.745038946 | -10.46645998 | 2.15E-19 | 4.68E-18 | 33.42280775 |
| TXK | -1.000341617 | 0.116373224 | -10.42245785 | 2.80E-19 | 6.03E-18 | 33.16219694 |
| EIF1AX | -1.007719703 | -0.544815441 | -10.41690068 | 2.90E-19 | 6.22E-18 | 33.1292937 |
| CLEC2L | 1.199505873 | -3.346442952 | 10.37854215 | 3.64E-19 | 7.75E-18 | 32.90224122 |
| FNBP4 | -1.003582255 | 0.511512361 | -10.3781418 | 3.65E-19 | 7.75E-18 | 32.89987205 |
| LELP1 | 1.168744313 | -2.192908427 | 10.35115487 | 4.29E-19 | 8.98E-18 | 32.74019891 |
| SENP6 | -1.253142442 | -2.119985628 | -10.34296187 | 4.51E-19 | 9.42E-18 | 32.69173479 |
| KRTAP5-7 | 1.062224563 | -3.188810501 | 10.33222618 | 4.81E-19 | 9.97E-18 | 32.62823786 |
| TMCC3 | -1.246554359 | -0.599434921 | -10.31120329 | 5.45E-19 | 1.12E-17 | 32.50392334 |
| SAMSN1 | -1.38476393 | 0.496167996 | -10.28946969 | 6.20E-19 | 1.27E-17 | 32.37544382 |
| CPNE6 | 1.14099304 | -2.531597192 | 10.28664556 | 6.31E-19 | 1.29E-17 | 32.3587516 |
| RGS9 | -1.35210677 | -2.133528447 | -10.2790452 | 6.60E-19 | 1.35E-17 | 32.31383254 |
| MAPK1IP1L | -1.096274658 | 0.60640783 | -10.26832301 | 7.04E-19 | 1.43E-17 | 32.25047116 |
| KLRF1 | -1.185908512 | 0.129061496 | -10.25870586 | 7.45E-19 | 1.51E-17 | 32.19364811 |
| KCNJ11 | 1.215672673 | -3.095123168 | 10.25311673 | 7.71E-19 | 1.55E-17 | 32.1606282 |
| PPP1R15B | -1.015054335 | -0.310186582 | -10.25148368 | 7.78E-19 | 1.56E-17 | 32.15098084 |
| TOR1AIP1 | -1.13171193 | -0.261853233 | -10.24024808 | 8.32E-19 | 1.66E-17 | 32.08461188 |
| CRYBB3 | 1.18947441 | -1.360673198 | 10.22443484 | 9.15E-19 | 1.80E-17 | 31.99122097 |
| GZMB | -1.243667486 | 4.349650151 | -10.21346432 | 9.76E-19 | 1.91E-17 | 31.92644317 |
| CTCF | -1.212538544 | -1.244721258 | -10.18989347 | 1.12E-18 | 2.18E-17 | 31.78729963 |
| ACTL6B | 1.023593976 | -1.555547152 | 10.1888845 | 1.13E-18 | 2.19E-17 | 31.78134452 |
| PNPLA5 | 1.243101425 | -3.227766946 | 10.17887774 | 1.20E-18 | 2.31E-17 | 31.72228831 |
| S1PR1 | -1.332986811 | 2.593624451 | -10.17578163 | 1.22E-18 | 2.34E-17 | 31.704018 |
| OR8U1 | 1.282218726 | -3.603961631 | 10.11661569 | 1.74E-18 | 3.27E-17 | 31.35504379 |
| NRF1 | -1.035685922 | -3.543932159 | -10.0887153 | 2.05E-18 | 3.78E-17 | 31.19059339 |
| ENO4 | 1.023236177 | -1.106609825 | 10.06671952 | 2.34E-18 | 4.27E-17 | 31.06099805 |
| SPON2 | -1.342583881 | 1.768496385 | -10.03065886 | 2.90E-18 | 5.23E-17 | 30.8486361 |
| ACTL8 | 1.01906096 | -2.874097751 | 10.02824868 | 2.94E-18 | 5.29E-17 | 30.83444701 |
| SNIP1 | -1.094192983 | -2.608809788 | -9.993236231 | 3.62E-18 | 6.40E-17 | 30.62838943 |
| ABT1 | -1.019394971 | -1.064893085 | -9.938503964 | 5.02E-18 | 8.68E-17 | 30.30652532 |
| RNASE11 | 1.16506235 | -3.898098963 | 9.898131368 | 6.38E-18 | 1.08E-16 | 30.06930702 |
| LPIN1 | -1.029374323 | 0.72805745 | -9.878637221 | 7.16E-18 | 1.21E-16 | 29.95482731 |
| ELMOD2 | -1.09821956 | -3.405907589 | -9.857411039 | 8.12E-18 | 1.35E-16 | 29.83022332 |
| MYL2 | 1.590662086 | -3.595395241 | 9.847469068 | 8.61E-18 | 1.44E-16 | 29.77187807 |
| ANKRD54 | -1.578295718 | -3.307123818 | -9.834848905 | 9.28E-18 | 1.54E-16 | 29.69783145 |
| SCAI | -1.054503143 | -1.341543311 | -9.834040384 | 9.33E-18 | 1.55E-16 | 29.6930882 |
| IKZF5 | -1.603333453 | -3.447140998 | -9.781220808 | 1.28E-17 | 2.06E-16 | 29.38337846 |
| MAP4K5 | -1.153018092 | -0.766819301 | -9.770397381 | 1.36E-17 | 2.18E-16 | 29.31995435 |
| CENPP | -1.234579957 | -2.210519644 | -9.760103421 | 1.45E-17 | 2.31E-16 | 29.25964552 |
| OTP | 1.176187251 | -3.647698704 | 9.759433585 | 1.45E-17 | 2.31E-16 | 29.2557216 |
| CALHM3 | 1.088066281 | -2.724214195 | 9.749651472 | 1.54E-17 | 2.43E-16 | 29.19842377 |
| IL13RA1 | 1.065098689 | -0.00019067 | 9.747188003 | 1.56E-17 | 2.46E-16 | 29.183996 |
| METTL7B | 1.234915072 | -1.643555971 | 9.744112967 | 1.59E-17 | 2.50E-16 | 29.16598748 |
| MSL1 | -1.319815092 | -0.468716371 | -9.740324098 | 1.62E-17 | 2.55E-16 | 29.14380003 |
| CDH5 | 1.184804549 | -1.554544626 | 9.722453771 | 1.81E-17 | 2.80E-16 | 29.03917519 |
| OTC | 1.514800467 | -3.59977937 | 9.712024165 | 1.92E-17 | 2.96E-16 | 28.97813095 |
| TMEM53 | 1.021496882 | -1.472478787 | 9.706813145 | 1.98E-17 | 3.05E-16 | 28.94763588 |
| HNRNPA0 | -1.333503502 | -2.0453248 | -9.704356816 | 2.01E-17 | 3.08E-16 | 28.93326249 |
| ZBED6 | -1.191983881 | 0.335091758 | -9.700713163 | 2.05E-17 | 3.14E-16 | 28.91194273 |
| OLIG1 | 1.162634076 | -1.567128311 | 9.698525806 | 2.08E-17 | 3.18E-16 | 28.89914484 |
| CRIM1 | -1.297023922 | -3.867710366 | -9.69345989 | 2.14E-17 | 3.27E-16 | 28.86950715 |
| SRPX2 | 1.042043895 | -3.004470842 | 9.688397332 | 2.21E-17 | 3.35E-16 | 28.83989224 |
| CHAF1B | -1.070551278 | -1.808229878 | -9.645852753 | 2.84E-17 | 4.25E-16 | 28.59113983 |
| NOTCH2 | -1.232874847 | 0.306991109 | -9.628536846 | 3.15E-17 | 4.67E-16 | 28.48996061 |
| WEE1 | -1.257573226 | -4.125867746 | -9.616685619 | 3.37E-17 | 4.99E-16 | 28.42073407 |
| SERTAD1 | -1.064509511 | -1.271510692 | -9.608819497 | 3.54E-17 | 5.21E-16 | 28.37479558 |
| MAN1A1 | -1.323745067 | -0.914123371 | -9.607152465 | 3.57E-17 | 5.26E-16 | 28.36506105 |
| CDC42SE1 | -1.069282658 | 1.958994662 | -9.555561348 | 4.84E-17 | 7.01E-16 | 28.06397542 |
| OR3A3 | 1.210682528 | -3.715856444 | 9.547411056 | 5.08E-17 | 7.32E-16 | 28.01644212 |
| SSTR4 | 1.086680686 | -3.737120531 | 9.531759581 | 5.57E-17 | 7.93E-16 | 27.92518585 |
| TM6SF2 | 1.110439144 | -3.04581232 | 9.498011096 | 6.80E-17 | 9.53E-16 | 27.72852605 |
| ATIC | -1.112982072 | -0.708168865 | -9.491936426 | 7.04E-17 | 9.86E-16 | 27.69314397 |
| PTGER2 | -1.061594227 | 3.243589308 | -9.472186035 | 7.91E-17 | 1.09E-15 | 27.57814209 |
| TRPV6 | 1.016211056 | -3.466203512 | 9.449791935 | 9.03E-17 | 1.24E-15 | 27.44781151 |
| SOX13 | -1.103637591 | -2.508266121 | -9.445508174 | 9.26E-17 | 1.27E-15 | 27.42288855 |
| MAK16 | -1.026897035 | -0.543227243 | -9.443005074 | 9.39E-17 | 1.29E-15 | 27.40832667 |
| ADCYAP1R1 | 1.018582591 | -3.395232139 | 9.385923389 | 1.31E-16 | 1.75E-15 | 27.076492 |
| IL20 | 1.157365373 | -2.771793075 | 9.38047729 | 1.36E-16 | 1.81E-15 | 27.04485634 |
| SLFN13 | -1.000276328 | -2.91129468 | -9.37681411 | 1.39E-16 | 1.84E-15 | 27.02357983 |
| TBX2 | 1.143476431 | -2.174661031 | 9.356829208 | 1.56E-16 | 2.05E-15 | 26.90753766 |
| ACRV1 | 1.022506529 | -1.761578769 | 9.345724586 | 1.66E-16 | 2.18E-15 | 26.84308397 |
| TTC39A | -1.154883657 | -4.040048244 | -9.316352594 | 1.98E-16 | 2.56E-15 | 26.67269012 |
| KIR2DL4 | -1.321826444 | 3.048647662 | -9.313848634 | 2.01E-16 | 2.60E-15 | 26.65816999 |
| OR2AG2 | 1.077412937 | -2.818582484 | 9.30312489 | 2.14E-16 | 2.75E-15 | 26.59599505 |
| ABLIM3 | 1.009857451 | 0.776032978 | 9.29686658 | 2.22E-16 | 2.84E-15 | 26.55971811 |
| COL18A1 | 1.021213386 | 2.83902362 | 9.27266817 | 2.55E-16 | 3.25E-15 | 26.4195053 |
| FCN3 | 1.033528582 | -3.448337147 | 9.272150942 | 2.56E-16 | 3.25E-15 | 26.4165093 |
| FNTA | -1.2529631 | 0.584146881 | -9.271359058 | 2.57E-16 | 3.27E-15 | 26.41192245 |
| CNTN3 | 1.068694768 | -3.211462968 | 9.266210156 | 2.65E-16 | 3.36E-15 | 26.3821007 |
| INSRR | -1.172831607 | -4.284907376 | -9.261057356 | 2.73E-16 | 3.45E-15 | 26.35226044 |
| THAP6 | -1.141349757 | -1.632160126 | -9.244066755 | 3.02E-16 | 3.79E-15 | 26.25389549 |
| RNMT | -1.127149276 | -0.228037762 | -9.239618093 | 3.10E-16 | 3.89E-15 | 26.22814789 |
| SAP30BP | -1.258119489 | 0.325921039 | -9.232419767 | 3.23E-16 | 4.04E-15 | 26.18649251 |
| CRYGA | 1.06811153 | -3.842356037 | 9.229914974 | 3.28E-16 | 4.09E-15 | 26.17199962 |
| ZNF26 | -1.051824329 | -2.130431095 | -9.228163472 | 3.31E-16 | 4.13E-15 | 26.16186591 |
| FCGR3A | -1.156506162 | 2.061194984 | -9.225744528 | 3.36E-16 | 4.18E-15 | 26.14787135 |
| PAK2 | -2.278419763 | -2.418343205 | -9.216867352 | 3.54E-16 | 4.38E-15 | 26.09652115 |
| NHSL1 | -1.432912711 | -2.905218543 | -9.204470068 | 3.81E-16 | 4.67E-15 | 26.02482953 |
| ZNF571 | -1.168866944 | -1.64752659 | -9.196735036 | 3.98E-16 | 4.86E-15 | 25.98011129 |
| ERCC5 | -1.085408837 | 1.97717658 | -9.189190539 | 4.16E-16 | 5.06E-15 | 25.93650371 |
| EIF4E2 | -1.159840344 | -0.338791346 | -9.189082503 | 4.17E-16 | 5.06E-15 | 25.93587933 |
| ENPP5 | -1.222975349 | -1.905716483 | -9.165030221 | 4.79E-16 | 5.77E-15 | 25.79691692 |
| PKP4 | -1.000222971 | -2.679142883 | -9.155257225 | 5.08E-16 | 6.07E-15 | 25.74047984 |
| ZNF25 | -1.069414806 | -4.383390733 | -9.12771184 | 5.96E-16 | 7.03E-15 | 25.58149413 |
| WTAP | -1.095410159 | -1.320038127 | -9.097218258 | 7.12E-16 | 8.34E-15 | 25.40563714 |
| CLEC3B | 1.053963048 | -3.787209023 | 9.077433124 | 7.99E-16 | 9.28E-15 | 25.29161849 |
| KIR3DL1 | -1.265446845 | 4.382711731 | -9.059700949 | 8.86E-16 | 1.02E-14 | 25.18948652 |
| RFX4 | 1.066517835 | -4.239333181 | 9.017182807 | 1.14E-15 | 1.29E-14 | 24.94481245 |
| NAP1L5 | -1.062344017 | -1.445487354 | -9.006291653 | 1.21E-15 | 1.37E-14 | 24.88218838 |
| ERGIC2 | -1.01641395 | -0.595647395 | -8.998530322 | 1.27E-15 | 1.43E-14 | 24.83757333 |
| B3GNT7 | -1.522607139 | -2.800672499 | -8.998069608 | 1.27E-15 | 1.43E-14 | 24.83492529 |
| BTN3A2 | -1.052553521 | 0.993811701 | -8.971846672 | 1.48E-15 | 1.65E-14 | 24.68426589 |
| TREH | 1.638975208 | -3.349747483 | 8.944998463 | 1.73E-15 | 1.91E-14 | 24.53013965 |
| BRS3 | 1.215571505 | -3.45151497 | 8.902953012 | 2.21E-15 | 2.41E-14 | 24.28903033 |
| DSTN | -1.011776105 | -0.948559207 | -8.893755582 | 2.33E-15 | 2.53E-14 | 24.23633038 |
| OR5A2 | 1.430105009 | -3.165462143 | 8.890186398 | 2.37E-15 | 2.58E-14 | 24.21588362 |
| G3BP2 | -1.112032208 | -0.059749799 | -8.868782973 | 2.69E-15 | 2.88E-14 | 24.09331899 |
| RNF183 | -1.046425487 | -3.713457544 | -8.86211579 | 2.79E-15 | 2.99E-14 | 24.05515726 |
| KLHDC2 | -1.110140674 | -0.47785025 | -8.846582876 | 3.06E-15 | 3.25E-14 | 23.96628167 |
| PRR20B | -1.431026224 | -0.019033207 | -8.842850125 | 3.12E-15 | 3.32E-14 | 23.94493047 |
| RBPJL | -2.322290244 | 2.524810474 | -8.838679474 | 3.20E-15 | 3.39E-14 | 23.92107757 |
| CDC42SE2 | -1.197031991 | 2.858983651 | -8.838171207 | 3.21E-15 | 3.40E-14 | 23.91817089 |
| FCRL6 | -1.13009043 | 3.024397712 | -8.832981196 | 3.31E-15 | 3.49E-14 | 23.88849308 |
| FLNA | -1.350874868 | -2.801085638 | -8.831872825 | 3.33E-15 | 3.51E-14 | 23.88215579 |
| JAKMIP1 | -1.029119127 | 0.694442068 | -8.824777159 | 3.47E-15 | 3.65E-14 | 23.84159061 |
| IFNA4 | 1.044912306 | -1.202637843 | 8.814275432 | 3.69E-15 | 3.86E-14 | 23.78157074 |
| RORB | -1.36387046 | -1.213768387 | -8.808708943 | 3.81E-15 | 3.98E-14 | 23.74976538 |
| ZDHHC6 | -1.235957402 | -0.9904984 | -8.797961603 | 4.05E-15 | 4.20E-14 | 23.68837473 |
| SFR1 | -1.100652083 | -0.047447351 | -8.766484585 | 4.86E-15 | 4.96E-14 | 23.50869957 |
| HIF1A | -1.258522168 | 1.015244843 | -8.766455035 | 4.86E-15 | 4.96E-14 | 23.50853098 |
| PROM1 | 1.077005237 | -4.182642574 | 8.688773016 | 7.61E-15 | 7.53E-14 | 23.06593604 |
| LGR6 | -1.23176452 | -1.250003936 | -8.685978927 | 7.73E-15 | 7.63E-14 | 23.05003887 |
| CCNDBP1 | -1.31534533 | 1.316834495 | -8.676929402 | 8.14E-15 | 7.98E-14 | 22.99856167 |
| DNAJC27 | -1.084658208 | -1.028001961 | -8.65873616 | 9.04E-15 | 8.80E-14 | 22.89512127 |
| PLK2 | -1.299667431 | -2.504337496 | -8.651560698 | 9.43E-15 | 9.16E-14 | 22.85434248 |
| CD300A | -1.670482563 | -0.436303644 | -8.638793369 | 1.01E-14 | 9.80E-14 | 22.78181045 |
| TBC1D23 | -1.311270919 | -1.590896934 | -8.633247236 | 1.05E-14 | 1.01E-13 | 22.75031286 |
| OR2T6 | 1.040883214 | -4.153459634 | 8.619153714 | 1.14E-14 | 1.09E-13 | 22.67030126 |
| PAFAH1B2 | -1.010604961 | -3.38418478 | -8.599537312 | 1.27E-14 | 1.21E-13 | 22.55900326 |
| AFAP1L1 | 1.158881305 | -3.731645743 | 8.593560812 | 1.32E-14 | 1.25E-13 | 22.52511008 |
| DEFB107A | 1.156038145 | -2.504980208 | 8.568617692 | 1.52E-14 | 1.42E-13 | 22.38373615 |
| DLEU7 | -1.583390519 | -2.607314354 | -8.565335528 | 1.55E-14 | 1.45E-13 | 22.36514303 |
| CRBN | -1.180228705 | 2.574725395 | -8.557537774 | 1.62E-14 | 1.51E-13 | 22.32097863 |
| TFDP3 | -1.368865069 | -0.878230344 | -8.554794941 | 1.64E-14 | 1.53E-13 | 22.30544701 |
| PAPSS2 | -1.074480529 | -3.518819701 | -8.495105667 | 2.31E-14 | 2.09E-13 | 21.96784574 |
| NCAM1 | -1.150743198 | -2.974207123 | -8.478997968 | 2.54E-14 | 2.28E-13 | 21.87687225 |
| OR5H1 | 1.238175219 | -3.548254697 | 8.47586642 | 2.58E-14 | 2.32E-13 | 21.85919236 |
| KIR2DS2 | -1.221741344 | 1.801731826 | -8.456713951 | 2.88E-14 | 2.57E-13 | 21.75110922 |
| ZFP37 | -1.018784862 | -4.407292214 | -8.44385899 | 3.10E-14 | 2.74E-13 | 21.67860998 |
| RIOK2 | -1.081706148 | -3.533627503 | -8.407492438 | 3.82E-14 | 3.35E-13 | 21.4737087 |
| TMEM144 | 1.274402952 | -2.276495917 | 8.39121893 | 4.19E-14 | 3.66E-13 | 21.38211406 |
| TRIAP1 | -1.223661903 | -1.622070777 | -8.378446463 | 4.51E-14 | 3.91E-13 | 21.31026668 |
| RRN3 | -1.069881731 | -2.786010292 | -8.366940112 | 4.81E-14 | 4.14E-13 | 21.24557309 |
| STXBP5 | -1.197822293 | -1.949466107 | -8.36013506 | 5.00E-14 | 4.30E-13 | 21.20732637 |
| CMYA5 | 1.03590668 | -2.937130886 | 8.346915965 | 5.39E-14 | 4.61E-13 | 21.1330608 |
| CDK5RAP1 | -1.1162482 | 0.632458312 | -8.31545826 | 6.45E-14 | 5.45E-13 | 20.9564914 |
| HIRA | -1.037229429 | -1.722655684 | -8.296822748 | 7.17E-14 | 6.00E-13 | 20.85200016 |
| GNL2 | -1.009251554 | 1.885659696 | -8.277299315 | 8.01E-14 | 6.66E-13 | 20.74261743 |
| HIGD1A | -1.379365836 | -2.285708057 | -8.271599445 | 8.27E-14 | 6.87E-13 | 20.71070004 |
| CRH | 1.07856989 | -4.253630928 | 8.260816197 | 8.79E-14 | 7.29E-13 | 20.65033843 |
| BNC2 | -1.625695967 | -2.177382435 | -8.255835066 | 9.05E-14 | 7.47E-13 | 20.62246477 |
| CREM | -1.218517436 | -1.98329077 | -8.253315741 | 9.18E-14 | 7.57E-13 | 20.60836925 |
| MLLT1 | -1.365127678 | -2.067370493 | -8.231980055 | 1.04E-13 | 8.52E-13 | 20.48905765 |
| JAKMIP3 | 1.119312458 | -1.974641731 | 8.218581464 | 1.12E-13 | 9.14E-13 | 20.41418698 |
| FLG | -1.23694437 | 0.981279889 | -8.216323995 | 1.13E-13 | 9.25E-13 | 20.4015766 |
| TPPP | -1.022187648 | -3.352451784 | -8.21028868 | 1.17E-13 | 9.55E-13 | 20.36786898 |
| KRT33B | -1.712479451 | -3.437788764 | -8.201280829 | 1.23E-13 | 1.00E-12 | 20.31757594 |
| CHI3L2 | -1.021425967 | -3.30320048 | -8.197853192 | 1.26E-13 | 1.02E-12 | 20.29844378 |
| CSTF2T | -1.050700068 | -1.737130864 | -8.188453038 | 1.33E-13 | 1.07E-12 | 20.24598926 |
| RHOV | 1.001590979 | -3.595768472 | 8.135512029 | 1.79E-13 | 1.43E-12 | 19.95097387 |
| ZNF430 | -1.196308538 | 4.041825097 | -8.100768353 | 2.17E-13 | 1.72E-12 | 19.75774185 |
| RASEF | 1.22118578 | -3.636125804 | 8.09223989 | 2.28E-13 | 1.80E-12 | 19.71035593 |
| DLG5 | -1.048208656 | -3.384782741 | -8.088404827 | 2.33E-13 | 1.83E-12 | 19.6890535 |
| PHLDA1 | -1.058012735 | -3.839789717 | -8.076951108 | 2.49E-13 | 1.95E-12 | 19.62545432 |
| IGFBP3 | -1.225478745 | -2.301222442 | -8.064690556 | 2.66E-13 | 2.07E-12 | 19.55741201 |
| SCN8A | -1.056366903 | -3.90285921 | -7.980584812 | 4.27E-13 | 3.25E-12 | 19.09169518 |
| ZNF365 | -1.133618259 | -4.278580597 | -7.9536744 | 4.97E-13 | 3.76E-12 | 18.94307552 |
| SH2D4A | -1.759571727 | -1.048271534 | -7.94280916 | 5.28E-13 | 3.97E-12 | 18.88312391 |
| RAB3IL1 | 1.024727803 | -3.861739051 | 7.924963998 | 5.84E-13 | 4.36E-12 | 18.78472734 |
| RAB2A | -1.084965357 | 2.092700729 | -7.875538193 | 7.69E-13 | 5.66E-12 | 18.51264643 |
| ZNF83 | -1.161134728 | -1.7727196 | -7.874050495 | 7.75E-13 | 5.70E-12 | 18.5044672 |
| ZNF354A | -1.213654159 | -2.44498779 | -7.837212325 | 9.52E-13 | 6.91E-12 | 18.3021281 |
| PPP4R1 | -1.003200299 | -1.627375236 | -7.801134247 | 1.16E-12 | 8.36E-12 | 18.10432846 |
| TSPAN9 | 1.267020254 | 0.261949669 | 7.783691326 | 1.28E-12 | 9.16E-12 | 18.00882763 |
| ZNF595 | -1.111836261 | 2.655410947 | -7.782155541 | 1.29E-12 | 9.22E-12 | 18.00042324 |
| DEFB134 | 1.006916831 | -3.454589494 | 7.776765164 | 1.33E-12 | 9.48E-12 | 17.97093035 |
| RXFP2 | 1.083234933 | -3.517160929 | 7.774136763 | 1.35E-12 | 9.59E-12 | 17.95655232 |
| FRG2C | 1.123719039 | -4.162281426 | 7.751857888 | 1.53E-12 | 1.08E-11 | 17.83475987 |
| KIR2DL5A | -1.23393048 | 1.029269022 | -7.744501551 | 1.59E-12 | 1.12E-11 | 17.79457587 |
| CAPZA3 | 1.152076022 | -3.694233752 | 7.743834639 | 1.60E-12 | 1.12E-11 | 17.79093363 |
| HOXD3 | 1.006395967 | -3.213648158 | 7.741956746 | 1.62E-12 | 1.13E-11 | 17.78067848 |
| TJP3 | -1.270200707 | 1.367603529 | -7.69434538 | 2.10E-12 | 1.45E-11 | 17.52101265 |
| OSBPL8 | -1.151782158 | -1.591090438 | -7.684730535 | 2.22E-12 | 1.53E-11 | 17.46865438 |
| KIR3DL2 | -1.073980051 | 2.961686712 | -7.671590152 | 2.39E-12 | 1.63E-11 | 17.39714125 |
| PRAMEF13 | 1.041830731 | -3.516694896 | 7.665213217 | 2.47E-12 | 1.68E-11 | 17.36245468 |
| PODN | -1.413320076 | -2.905426904 | -7.657820051 | 2.58E-12 | 1.75E-11 | 17.32225542 |
| TMED2 | -1.030264558 | 0.423155879 | -7.652744767 | 2.65E-12 | 1.79E-11 | 17.29466863 |
| MAL | -1.075686616 | 0.548674711 | -7.631936792 | 2.97E-12 | 1.99E-11 | 17.1816462 |
| COX18 | -1.01228357 | -3.338609012 | -7.591973219 | 3.70E-12 | 2.45E-11 | 16.96493847 |
| LHX9 | 1.069678944 | -4.123856446 | 7.581329306 | 3.93E-12 | 2.59E-11 | 16.90730137 |
| TNFSF9 | -1.125266671 | -1.823339242 | -7.573861551 | 4.09E-12 | 2.69E-11 | 16.8668837 |
| HBS1L | -1.284751938 | -3.185374876 | -7.568815671 | 4.21E-12 | 2.76E-11 | 16.8395835 |
| DNAJC8 | -1.138388314 | -3.622531388 | -7.561298744 | 4.38E-12 | 2.87E-11 | 16.7989283 |
| ZNF207 | -1.005676187 | 2.626666998 | -7.538945669 | 4.96E-12 | 3.23E-11 | 16.67813383 |
| ZNF680 | -1.04334371 | -3.375003601 | -7.495012803 | 6.31E-12 | 4.06E-11 | 16.44117139 |
| RNF224 | -1.03321499 | 0.978842908 | -7.458529875 | 7.70E-12 | 4.87E-11 | 16.24484815 |
| KIR2DL2 | -1.033608718 | 2.556643148 | -7.408593723 | 1.01E-11 | 6.30E-11 | 15.9768105 |
| GPIHBP1 | -1.088353103 | -0.534076167 | -7.394289828 | 1.09E-11 | 6.76E-11 | 15.9001793 |
| KRTAP19-2 | -1.033823134 | 1.796689838 | -7.294841255 | 1.88E-11 | 1.12E-10 | 15.36923231 |
| NUAK1 | -1.073350657 | -3.350998535 | -7.260558131 | 2.26E-11 | 1.34E-10 | 15.1869534 |
| CCL4 | -1.44529461 | 3.241936125 | -7.244525237 | 2.46E-11 | 1.45E-10 | 15.10184364 |
| RARB | -1.080167948 | -3.515724716 | -7.243095569 | 2.48E-11 | 1.46E-10 | 15.09425852 |
| PPP1R15A | -1.001108052 | 3.077718958 | -7.238105343 | 2.55E-11 | 1.50E-10 | 15.06778825 |
| SLC30A5 | -1.167664218 | -0.19959309 | -7.2174106 | 2.85E-11 | 1.65E-10 | 14.95810449 |
| RNF165 | -1.072883506 | -3.40736438 | -7.208865962 | 2.98E-11 | 1.73E-10 | 14.91285966 |
| MDFIC | -1.131704742 | -2.187621281 | -7.191406164 | 3.28E-11 | 1.89E-10 | 14.82048551 |
| PDE6H | -1.347563259 | -3.003440181 | -7.171143523 | 3.65E-11 | 2.09E-10 | 14.71341344 |
| TNFAIP3 | -1.191080241 | 3.026448827 | -7.152407417 | 4.04E-11 | 2.30E-10 | 14.6145339 |
| AMPH | 1.269765948 | -2.45112367 | 7.128797531 | 4.59E-11 | 2.60E-10 | 14.49010654 |
| ACOT12 | 1.058477344 | -3.34042621 | 7.115052235 | 4.94E-11 | 2.78E-10 | 14.41775655 |
| ST6GAL2 | 1.061420662 | 2.18327704 | 7.10536124 | 5.20E-11 | 2.91E-10 | 14.36678668 |
| PHACTR1 | -1.30630245 | -2.842297432 | -6.988622706 | 9.69E-11 | 5.19E-10 | 13.75542455 |
| PASK | -1.036368176 | -1.920093684 | -6.983024631 | 9.98E-11 | 5.34E-10 | 13.72623075 |
| HBQ1 | 1.165987826 | 0.675553033 | 6.873520074 | 1.78E-10 | 9.17E-10 | 13.15749212 |
| AMD1 | -1.062243707 | 0.062925764 | -6.82104579 | 2.35E-10 | 1.19E-09 | 12.8865476 |
| CUL3 | -1.019809581 | -2.682888637 | -6.782396391 | 2.88E-10 | 1.43E-09 | 12.68765936 |
| INSL6 | 1.017321321 | -2.743302748 | 6.778994713 | 2.93E-10 | 1.46E-09 | 12.67018205 |
| ZNF208 | -1.019971181 | 2.585383794 | -6.607869027 | 7.14E-10 | 3.35E-09 | 11.79684287 |
| CD80 | -1.089293192 | -3.787452119 | -6.534488265 | 1.04E-09 | 4.77E-09 | 11.4259591 |
| ZNF728 | -1.286325942 | 3.011595291 | -6.473056244 | 1.43E-09 | 6.41E-09 | 11.11718319 |
| PAGE1 | 1.069056111 | -4.393796014 | 6.360403579 | 2.54E-09 | 1.11E-08 | 10.55511261 |
| ANGPTL2 | -1.134830656 | 2.328123171 | -6.335378755 | 2.88E-09 | 1.25E-08 | 10.43099666 |
| SYNM | -1.040962005 | -3.651768702 | -6.245155768 | 4.54E-09 | 1.93E-08 | 9.985805007 |
| TM4SF19 | -1.251222834 | -1.905900569 | -6.240040848 | 4.66E-09 | 1.98E-08 | 9.960674668 |
| OSM | -1.520632514 | -1.559346067 | -6.196783616 | 5.79E-09 | 2.43E-08 | 9.748616669 |
| PZP | -1.296764405 | -0.815303967 | -6.049494444 | 1.20E-08 | 4.84E-08 | 9.032991986 |
| TRAPPC6B | -1.062801708 | -1.409813419 | -5.954748342 | 1.92E-08 | 7.54E-08 | 8.57803243 |
| SERPINB2 | -1.44097688 | -0.821168242 | -5.880183324 | 2.76E-08 | 1.06E-07 | 8.22301919 |
| TRIM61 | -1.016047319 | -0.278793872 | -5.75744249 | 5.00E-08 | 1.85E-07 | 7.644605111 |
| ACTG2 | 1.055291947 | -1.828712557 | 5.742270544 | 5.38E-08 | 1.99E-07 | 7.573632255 |
| IER3 | -1.224301157 | -0.523807624 | -5.676753034 | 7.36E-08 | 2.66E-07 | 7.268495002 |
| EGR3 | -1.217130119 | -1.259184982 | -5.441819776 | 2.23E-07 | 7.57E-07 | 6.192765902 |
| PRTN3 | 1.020606117 | -3.099961409 | 5.308785116 | 4.12E-07 | 1.35E-06 | 5.596830654 |
| IL1B | -1.635823861 | 1.223604668 | -5.237521181 | 5.70E-07 | 1.84E-06 | 5.281659898 |
| KIR2DS3 | -1.033571125 | 1.301688484 | -5.150238815 | 8.45E-07 | 2.66E-06 | 4.899586945 |
| IL1A | -2.033268339 | -3.263062176 | -4.838505768 | 3.34E-06 | 9.76E-06 | 3.571610112 |
| SULT6B1 | 1.104696143 | -3.677092253 | 4.805411703 | 3.86E-06 | 1.11E-05 | 3.434089923 |
| CXCL3 | -1.246882295 | -3.519911875 | -4.706219661 | 5.89E-06 | 1.67E-05 | 3.026001334 |
| CXCL2 | -1.247787677 | -0.213352381 | -4.662625417 | 7.08E-06 | 1.99E-05 | 2.848615129 |
| CCL20 | -1.506197574 | -2.849662018 | -3.736318586 | 0.000268983 | 0.000607907 | -0.617363022 |
| HES1 | -1.044370747 | -2.309611248 | -3.494994881 | 0.000631281 | 0.001350217 | -1.418455772 |
| MTRNR2L8 | -1.294939583 | 6.1829114 | -3.315362394 | 0.001159619 | 0.002385282 | -1.985454234 |
| CXCL1 | -1.016004217 | -2.846135575 | -3.308333314 | 0.001186979 | 0.00243718 | -2.007122149 |
| G0S2 | -1.137122242 | 2.025288316 | -3.140564116 | 0.002048775 | 0.004057196 | -2.512501628 |
| CCL3L3 | -1.099897846 | 2.7042633 | -2.998088828 | 0.003203928 | 0.006124673 | -2.923669912 |

Supplementary Figure 1. (A) Hierarchical cluster trees and (B) heatmap of clinical traits (branches represent samples and ordinate represents the height of hierarchical clusters. The branch refers to a red clinical trait representing sample pertaining to such a trait).
